# Exosomal miR-106a-5p from highly metastatic colorectal cancer cells drives liver metastasis by inducing macrophage M2 polarization in the tumor microenvironment

**DOI:** 10.1186/s13046-024-03204-7

**Published:** 2024-10-09

**Authors:** Yahang Liang, Junyu Li, Yuli Yuan, Houqiong Ju, Hualin Liao, Mingming Li, Yang Liu, Yao Yao, Lingling Yang, Taiyuan Li, Xiong Lei

**Affiliations:** 1 Department of General surgery, The First Affiliated Hospital, Jiangxi Medical College, Nanchang University, Nanchang, Jiangxi 330006 China; 2 Gastrointestinal Surgical Institute, Nanchang University, Nanchang, Jiangxi 330006 China; 3 Department of Orthopedics, The First Affiliated Hospital, Jiangxi Medical College, Nanchang University, Nanchang, Jiangxi 330006 China; 4 Department of Gastroenterology, The Second Affiliated Hospital, Jiangxi Medical College, Nanchang University, Nanchang, Jiangxi 330006 China

**Keywords:** Exosomes, miRNAs, Macrophage, Colorectal cancer, Liver metastasis

## Abstract

**Background:**

The tumor microenvironment (TME) is a dynamic system orchestrated by intricate cell-to-cell crosstalk. Specifically, macrophages within the TME play a crucial role in driving tumor progression. Exosomes are key mediators of communication between tumor cells and the TME. However, the mechanisms underlying exosome-driven crosstalk between tumor cells and macrophages during colorectal cancer (CRC) progression remain incompletely elucidated.

**Methods:**

Single-cell RNA sequencing were analyzed using the Seurat package. Exosomes were isolated using ultracentrifugation and characterized by transmission electron microscopy, nanoparticle tracking analysis, and western blot. miRNAs differentially expressed in exosomes were analyzed using the limma package. CD206 expression in CRC tissues, exosomes tracing, and exosomal miR-106a-5p transport were observed through immunofluorescence. Macrophage polarization was assessed via qRT-PCR, ELISA, and flow cytometry. The interactions between miR-106a-5p, hnRNPA1, and SOCS6 were evaluated using miRNA pull-down, RIP, and dual-luciferase reporter assays. Transwell assays and liver metastasis model explored the role of exosomal miR-106a-5p-induced M2 macrophages in promoting CRC liver metastasis.

**Result:**

The proportion of M2 macrophages is increased in CRC with liver metastasis compared to those without. Highly metastatic CRC cells release exosomes enriched with miR-106a-5p, which promote macrophages M2 polarization by suppressing SOCS6 and activating JAK2/STAT3 pathway. These M2 macrophages reciprocally enhance CRC liver metastasis. hnRNPA1 regulate the transport of miR-106a-5p into exosomes. Clinically, elevated miR-106a-5p in plasma exosomes correlated with liver metastasis and poor prognosis.

**Conclusion:**

CRC-derived exosomal miR-106a-5p plays a critical role in promoting liver metastasis and is a potential biomarker for the prevention and treatment of CRC liver metastasis.

**Supplementary Information:**

The online version contains supplementary material available at 10.1186/s13046-024-03204-7.

## Background

Distant metastasis stands as a critical determinant of mortality in colorectal cancer (CRC) mortality [1]. Due to the unique anatomical characteristics of portal vein circulation in the colorectum, more than 50% of CRC patients experience liver metastasis during the course of their disease [2]. Upon the occurrence of liver metastasis, the 5-year overall survival (OS) rate for CRC patients plummets to a mere 20% [3]. Therefore, there is a pressing need to comprehensively investigate the intrinsic mechanisms driving CRC liver metastasis, pinpoint reliable molecular targets, and explore effective prevention and treatment strategies to enhance the prognosis of CRC patients with liver metastasis.

As our understanding of tumors deepens, a growing body of evidence underscores the significant impact of various cells interactions within the tumor microenvironment (TME) on tumor metastasis [4, 5]. The TME, comprising multiple non-tumor cells, forms a complex milieu that influences tumor malignancy, immune evasion, and patient’ response to pharmacotherapy and overall survival [6]. Among these non-tumor cells, tumor-associated macrophages (TAMs) emerge as crucial regulators role in tumor metastasis [7–9]. Current studies indicate that macrophages can be broadly induced into two distinct types: classically activated M1 macrophages (expressing marker genes such as CD86 and IL-1β) and alternatively activated M2 macrophages (expressing marker genes such as CD163, CD206, IL-10, Arginase-1, and TGF-β) [10]. CD163 is a membrane glycoprotein that belongs to the scavenger receptor family and is predominantly expressed on M2 macrophages. CD206, also known as the mannose receptor C-type 1 (MRC1), is also a typical marker of M2 macrophages [11]. The increase in M2 macrophages is closely linked to tumor metastasis, as these macrophages secrete anti-inflammatory cytokines such as IL-10 and TGF-β, participating in immune regulation, wound healing, angiogenesis, and promoting tumor progression [12]. Therefore, when TAMs in the TME undergo M2 polarization, the microenvironment becomes more conducive to tumor growth, significantly amplifying tumor metastasis.

Exosomes, membranous vesicles with a diameter of approximately 30-150nm, are secreted by various cells [13]. Serving as cellular messengers, exosomes transport a variety of bioactive molecules, including nucleotides, proteins, and lipids, facilitating intercellular communication and participating in the regulation of numerous physiological and pathological processes [14]. MicroRNAs (miRNAs), non-coding RNAs measuring 20–24 nucleotides in length, exert diverse biological functions by binding to the 3’ UTR regions of target genes and regulating their expression [15]. Studies report that 43% of RNA in exosomes comprises miRNA, underscoring the significance of miRNA in exosomal function [16]. Moreover, several studies have shown that exosomal miRNAs can regulate macrophage M2 polarization, thereby promoting metastasis in cancers such as prostate cancer, glioma and lung cancers [17–19]. However, the role and mechanism of exosomal miRNAs in CRC remain inadequately explored.

Our research unveiled an increased proportion of M2 macrophages in CRC tissues with liver metastasis compared to those without. Subsequent investigations revealed that highly metastatic CRC cells release exosomes rich in miR-106a-5p, inducing M2 polarization in macrophages. Consequently, exosomal miR-106a-5p-induced M2 macrophages reciprocally enhance CRC liver metastasis. These findings contribute to the identification of a novel and specific biomarker for the prevention and treatment of CRC.

## Methods

### Patient samples and follow-up

A total of 311 plasma samples, tumor tissues, and paired adjacent non-tumorous tissues were obtained from CRC patients who underwent surgical resection at the First Affiliated Hospital of Nanchang University between September 2019 and August 2020. Additionally, plasma samples were collected from 40 CRC surgical patients both before and on the fifth day after surgery. For immunofluorescence analysis, 20 fresh CRC tissues each with and without liver metastasis were randomly selected from this cohort. Moreover, 124 CRC patients’ plasma specimens and 15 plasma specimens from healthy individuals were used for quantitative real-time polymerase chain reaction (qRT-PCR). Regular follow-up procedures, as detailed in our previous study described [4], were implemented. Approval for this study was obtained from the Ethics Committee of the First Affiliated Hospital of Nanchang University, and informed consent was obtained from each participant.

### Statistical analysis

Data analysis and visualization were performed using SPSS 22.0 (IBM, USA), GraphPad Prism 8.0 (GraphPad Software, USA), and R (Version 4.0.5) software. Difference between two groups were analyzed using χ^2^ test and Student’s t test. Analysis of variance (ANOVA) was used to compare the difference between multiple groups. Kaplan-Meier method and log-rank test analyzed the differences in OS and disease-free survival (DFS) of CRC patients. A significance level of *P* < 0.05 denoted statistical significance.

Additional materials and methods utilized in this study are exhibited in the supplementary materials.

## Results

### Single-cell RNA sequencing revealed increased infiltration of M2 macrophage in CRC with liver metastasis

**Fig. 1:**
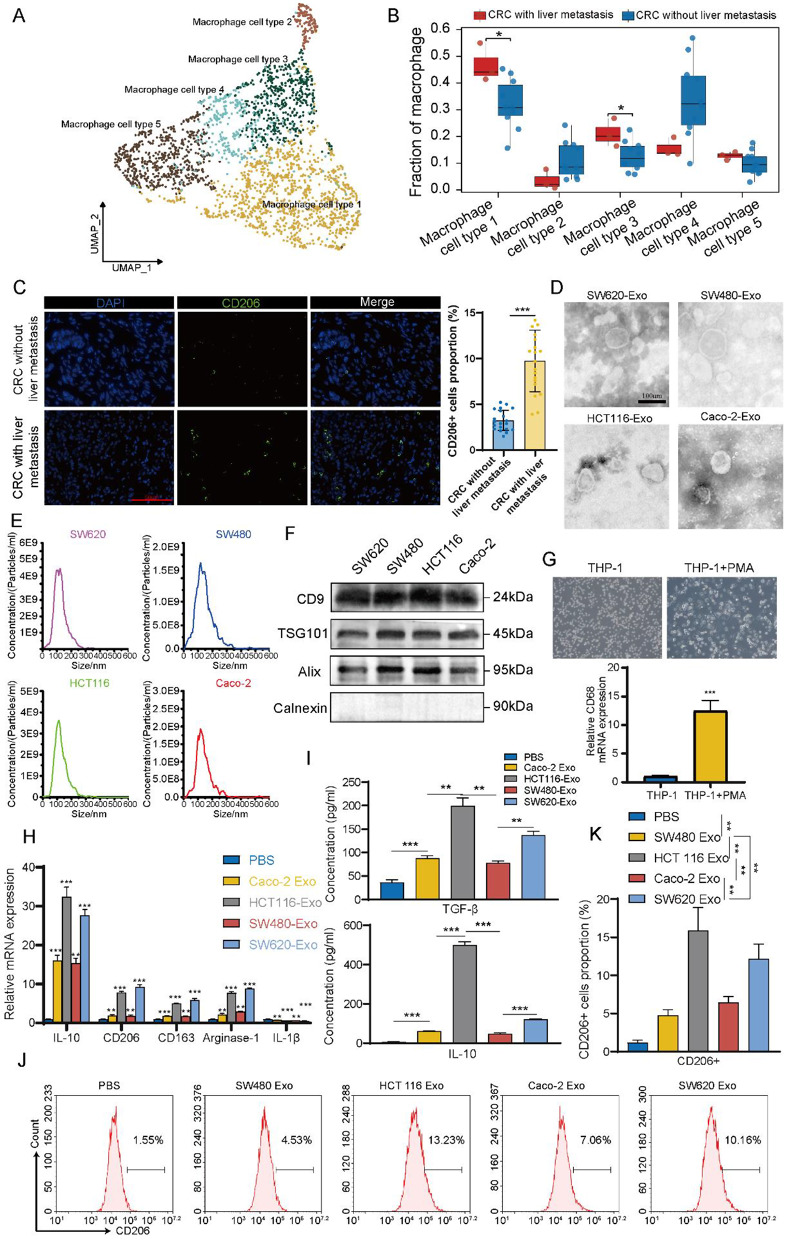
Exosomes released by highly metastatic CRC cells drive macrophages M2 polarization. **(A)** Uniform manifold approximation and projection (UMAP) plot of macrophage clusters. **(B)** Boxplot showed the proportions of macrophage types in CRC tissues with or without liver metastasis. **(C)** Immunofluorescence assay was used to detected the proportions of CD206^+^ cells in CRC tissues with (*n* = 20) or without (*n* = 20) liver metastasis. Scale bar = 50 μm. **(D)** TEM showed the typical structures of SW620, SW480, HCT 116 and Caco-2 exosomes. Scale bar = 100 μm. **(E)** The particle size of exosomes was detected by NTA. **(F)** The presence and absence exosomal markers were detected by western blot. **(G)** Representative image following 24 h treatment of THP-1 cells with 100 ng/mL phorbol 12-myristate 13-acetate (PMA) to induce their differentiation into Mφ. qRT-PCR was utilized to detect the marker gene expression of macrophage (CD68). **(H)** qRT-PCR was performed to detect changes in the expression of M2 (IL-10, CD206, CD163, and Arginase-1) and M1 (IL-1β) macrophages marker genes in Mφ after incubated with different exosomes. **(I)** ELISA was used to assess the secretion of TGF-β and IL-10 by Mφ treated with different exosomes. **J-K.** Flow cytometry was used to detect the proportion of CD206^+^ macrophages in Mφ after incubated with different exosomes. The data presented herein represent the outcomes of a minimum of three independent experiments and are depicted as the mean ± standard deviation (SD). * *P* < 0.05, ** *P* < 0.01, *** *P* < 0.001

To profile the differences in the primary TME between CRC tissues with liver metastasis and those without liver metastasis, we analyzed single-cell RNA sequencing data obtained from the Gene Expression Omnibus (GEO) database (GSE178318, GSE205506), comprising thirteen CRC tumors (three with liver metastasis and ten without). Following quality filtering, we obtained a total of 84,040 high-quality single cells for subsequent analysis. Cluster analysis of combined samples, based on classical markers, identified seven major cell types: T/NK cells, B/plasma cells, myeloid cells, epithelial cells, endothelial cells, fibroblasts cells, and mast cells (Figure S1A and S1B). In CRC tissues with liver metastasis, the proportion of T/NK cells (50.86% vs. 27.46%) and myeloid cells (13.38% vs. 6.74%) was higher compared to CRC tissues without liver metastasis (Figure S1C). Moreover, numerous studies indicate that myeloid cells are closely associated with the progression of CRC [20, 21]. Next, we extracted a total of 7,044 myeloid cells and re-clustered them into six subtypes (Figure S1D). In CRC tissues with liver metastasis, the proportion of macrophage cells (40.87% vs. 27.61%) was higher compared to CRC tissues without liver metastasis (Figure S1E). Therefore, we further re-clustered the macrophage cells into five subtypes (Fig. 1A) based on their gene expression profiles. We observed that macrophage cell type 1 exhibited high expression of CD163 and CD206 (also known as MRC1), suggesting that these cells possess characteristics of M2 macrophages (Figure S1F). Further analysis revealed that the proportion of macrophage cell type 1 (M2 macrophage) was significantly higher in CRC tissues with liver metastasis compared to those without liver metastasis (46.34% vs. 32.54%, *P* < 0.05) (Fig. 1B and S1G). These findings suggest that M2 macrophages are significantly more abundant in CRC tissues with liver metastasis compared to those without liver metastasis. Given that some studies have demonstrated an association between M2 macrophages and tumor metastasis [22, 23], we further investigated the relationship between M2 macrophages and CRC liver metastasis.

### Exosomes released by highly metastatic CRC cells drive macrophages M2 polarization

Initially, immunofluorescence assay was conducted to validate the results of single-cell RNA sequencing, revealing a significant increase in M2 macrophages in CRC tissues with liver metastasis compared to those without (Fig. 1C, *P* < 0.001). This suggests the presence of a substance in CRC tissues with liver metastasis that may promote M2 polarization of macrophage. Numerous studies have demonstrated that exosomes play a crucial role in regulating the TME [24, 25]. Accordingly, we selected two CRC cell lines with high metastatic potential (HCT 116 and SW620) and two with low metastatic potential (SW480 and Caco-2) for further study [26, 27] (metastatic capability shown in Figure S2). Transmission electron microscopy and nanoparticle tracking analysis revealed that the exosomes had diameters ranging from 30 to 150 nm (Fig. 1D and E). Western blot confirmed the presence of exosomal markers CD9, TSG101, and Alix, and the absence of the endoplasmic reticulum protein Calnexin (Fig. 1F). To explore the influence of exosomes derived from CRC cell lines with different metastatic potentials on macrophage polarization, we treated PMA-induced THP-1 cells (named as Mφ in subsequence, Fig. 1G) with exosomes from the above four CRC cell lines. qRT-PCR showed that exosomes from highly metastatic CRC cells induced higher expression levels of IL-10, CD206, CD163, and Arginase-1 in Mφ compared to those from low metastatic CRC cells (Fig. 1H). ELISA indicated that exosomes derived from highly metastatic CRC cells induced Mφ to secret more TGF-β and IL-10 (markers of M2 macrophages) compared to exosomes from low metastatic CRC cells (Fig. 1I). Flow cytometry also demonstrated a higher proportion of CD206^+^ macrophages when incubated with exosomes from highly metastatic CRC cells (Fig. 1J and K). These results indicate that highly metastatic CRC cells could induce more macrophages M2 polarization.

### miR-106a-5p abundance in exosomes from highly metastatic CRC cells

**Fig. 2:**
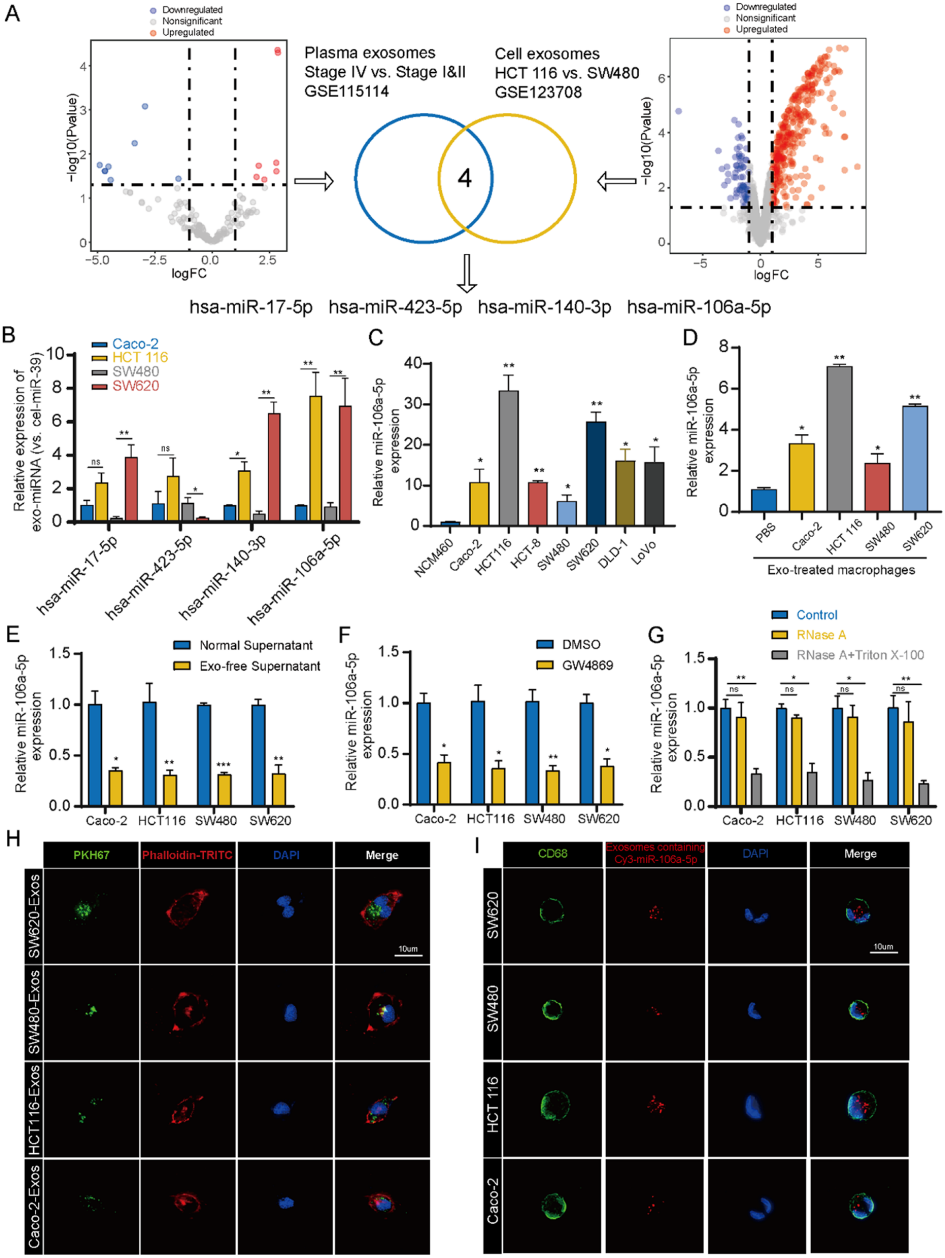
miR-106a-5p abundance in exosomes from highly metastatic CRC cells. **(A)** Volcano plot exhibiting the differentially expressed exosomal miRNAs in the GSE115114 and GSE123708. **(B)** qRT-PCR was performed to detect the expression of four miRNAs in exosomes derived from four CRC cells. **(C)** The expression of miR-106a-5p in NCM460 and CRC cell lines. **(D)** The expression of miR-106a-5p in Mφ after treated with PBS, Caco-2 exosomes, HCT 116 exosomes, SW480 exosomes or SW620 exosomes, respectively. **E-F.** The expression levels of miR-106a-5p in Mφ after incubated with physically (**E**) or pharmacologically (**F**) exosome-depleted supernatants compared to those treated with standard supernatant. **G.** The expression levels of miR-106a-5p were detected in the supernatant of CRC cells following treatment with RNase A or RNase A plus Triton X-100. **H.** The presence of green fluorescent signals in Mφ after treated with PKH67-labeled exosomes for 24 h. Scale bar = 10 μm. **I.** Mφ were incubated with exosomes derived from SW620/SW480/HCT 116/Caco-2 transfected with Cy3-labeled miR-106a-5p (red). Scale bar = 10 μm. The data presented herein represent the outcomes of a minimum of three independent experiments and are depicted as the mean ± standard deviation (SD). ns = No significant difference, * *P* < 0.05, ** *P* < 0.01, *** *P* < 0.001

The components in exosomes, especially miRNAs, play a crucial role in cell-to-cell crosstalk [28]. To clarify the exosomal miRNAs that regulate macrophages M2 polarization, we analyzed miRNA sequencing data from the GEO database (GSE115114, GSE123708). Four miRNAs were found to be highly expressed in exosomes from both the plasma of metastatic patients and HCT 116 cells (Fig. 2A). Subsequent qRT-PCR revealed that hsa-miR-106a-5p (named as miR-106a-5p in subsequence) showed the most significant expression difference in exosomes from high and low metastatic CRC cells (Fig. 2B). Further qRT-PCR assay on cells indicated that miR-106a-5p was highly expressed in CRC cells compared to normal colonic epithelial cells (NCM460), with even higher expression observed in highly metastatic CRC cells (Fig. 2C). Additionally, Mφ treated with exosomes from highly metastatic CRC cells showed significantly higher levels of miR-106a-5p than those treated with exosomes from low metastatic cells (Fig. 2D). To determine whether miR-106a-5p is transferred from CRC cells to macrophages via exosomes, Mφ were incubated with either standard supernatant or exosome-depleted supernatant from CRC cells. The expression of miR-106a-5p were significantly reduced in Mφ treated with physically (Fig. 2E) or pharmacologically (Fig. 2F) exosome-depleted supernatants compared to those treated with standard supernatant. Furthermore, under RNase A treatment, the expression of miR-106a-5p in the supernatant from CRC cells did not change, but significantly decreased with combined RNase A and Triton X-100 treatment, suggesting that extracellular miR-106a-5p is primarily encapsulated within vesicles, rather than being directly released from CRC cells (Fig. 2G). Additionally, Mφ were treated with PKH67-labeled exosomes. After 24 h, green fluorescent signals were observed in Mφ, indicating the internalization of PKH67-labeled exosomes (Fig. 2H). Concurrently, Mφ were incubated with exosomes derived from CRC cells transfected with Cy3-labeled miR-106a-5p. After 24 h, Cy3-labeled miR-106a-5p was observed in the Mφ, demonstrating the transfer of miR-106a-5p from CRC cells to macrophages (Fig. 2I). In summary, these results suggest that miR-106a-5p is highly expressed in exosomes from highly metastatic CRC cells and could be transferred to macrophages via exosomes.

### Regulation of miR-106a-5p packaging into exosomes by hnRNPA1

**Fig. 3:**
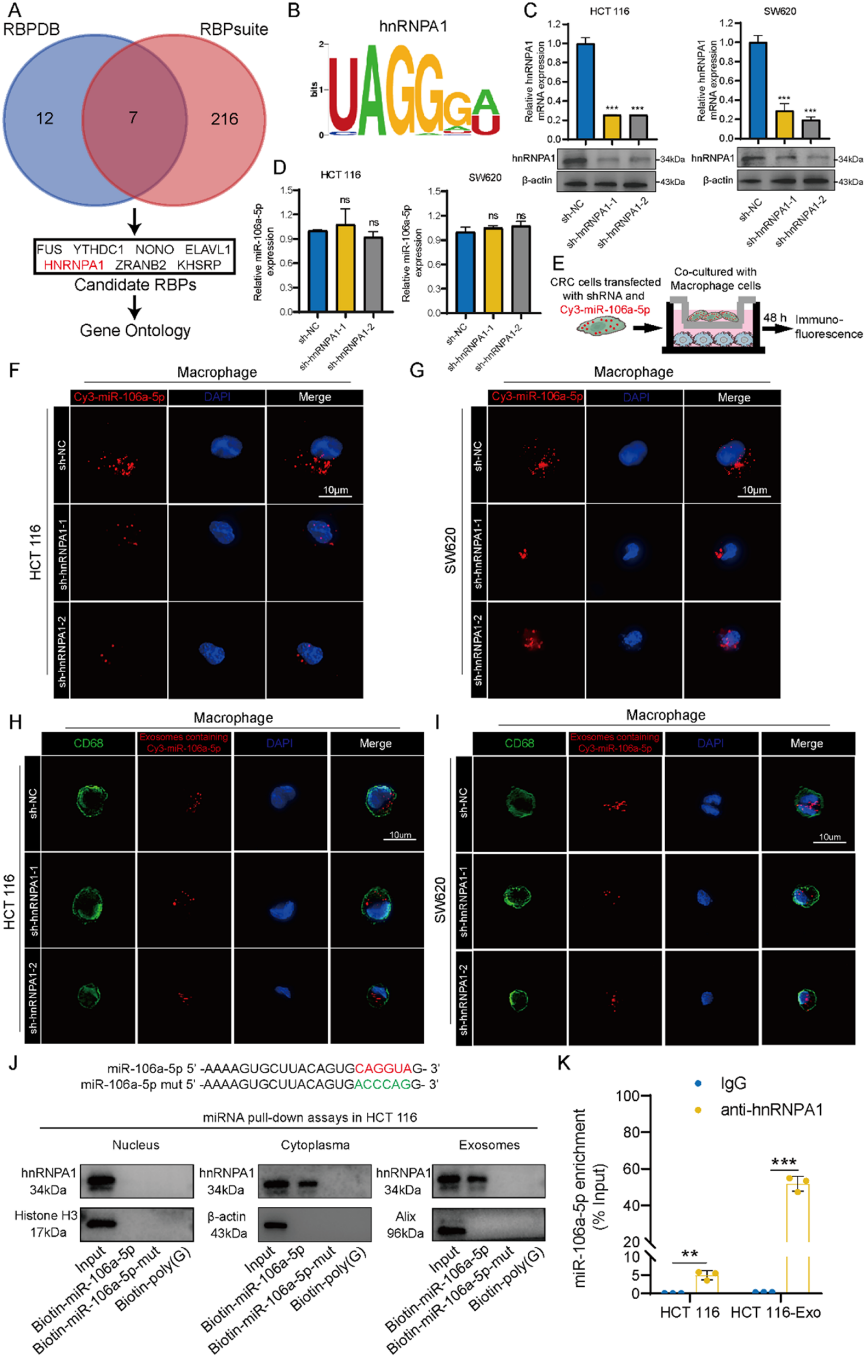
Regulation of miR-106a-5p packaging into exosomes by hnRNPA1. **(A)** The potential RBPs that may bind to miR-106a-5p. **(B)** The specific interaction between the miR-106a-5p sequence and hnRNPA1 motifs. **C-D.** After transfecting HCT 116 and SW620 cells with sh-hnRNPA1, the expression of hnRNPA1 was detected by qRT-PCR and western blot (**C**), and the expression of miR-106a-5p was detected by qRT-PCR (**D**). **E-G.** Mφ cells were co-cultured with SW620/HCT 116 pre-transfected with sh-hnRNPA1 and Cy3-miR-106a-5p (red). Immunofluorescence was performed to detect the red fluorescent signals in macrophages. Scale bar = 10 μm. **H-I.** Mφ cells were incubated with exosomes derived from SW620/HCT 116 transfected with sh-hnRNPA1 and Cy3-miR-106a-5p (red). Immunofluorescence was performed to detect the red fluorescent signals in macrophages. Scale bar = 10 μm. **J.** Western blot was employed to assess hnRNPA1 expression in samples obtained from miRNA pulldowns, utilizing nuclear, cytoplasmic, or exosomal lysates from HCT116 cells. **K.** RIP assay was executed using an anti-hnRNPA1 antibody (or IgG as a control) on lysates derived from HCT 116 cells or exosomes. qRT-PCR was used to quantify miR-106a-5p levels in the immunoprecipitated samples, expressed as percentages relative to the input (% input). The data presented herein represent the outcomes of a minimum of three independent experiments and are depicted as the mean ± standard deviation (SD). ns = No significant difference, ** *P* < 0.01, *** *P* < 0.001

Previous studies have shown that the transport of miRNA into exosomes requires the involvement of specific RNA-binding proteins (RBPs) [29]. To clarify the mechanism of miR-106a-5p transport into exosomes, we utilized the database of RNA-binding protein specificities (RBPDB, http://rbpdb.ccbr.utoronto.ca/) and RNA-binding protein site prediction (RBPsuite, http://www.csbio.sjtu.edu.cn/bioinf/RBPsuite/) to screen for RBPs that may bind to miR-106a-5p (Fig. 3A). A total of seven RBPs were in the intersection of the two databases. Gene Ontology analysis of the seven RBPs identified heterogeneous nuclear ribonucleoprotein A1 (hnRNPA1) as being localized in exosomes (Table S3), and hnRNPA1 was found to have a specific binding site for miR-106a-5p (Fig. 3B). Meanwhile, we found that knockdown of hnRNPA1 in CRC cells did not change the expression of miR-106a-5p (Fig. 3C and D). Co-culturing CRC cells transfected with sh-hnRNPA1 and Cy3-miR-106a-5p with macrophages showed that knocking down hnRNPA1 in CRC cells significantly reduced the amount of miR-106a-5p transported to macrophages via exosomes (Fig. 3E-G). Additionally, after extracting exosomes from CRC cells transfected with sh-hnRNPA1 and Cy3-miR-106a-5p and incubating them with macrophages, immunofluorescence also showed that knocking down hnRNPA1 in CRC cells significantly reduced the transfer of miR-106a-5p to macrophages via exosomes (Fig. 3H-I), indicating that hnRNPA1 regulates miR-106a-5p encapsulation into exosomes. Additionally, miRNA pull-down and RIP experiments demonstrated that miR-106a-5p and hnRNPA1 interact in the cytoplasm and exosomes of HCT 116 and SW620 cells, but not in the nucleus. Mutation of the miR-106a-5p binding sequence (CAGGUA) eliminated this interaction (Fig. 3J and K; Figure S3A and S3B). These results suggest that in CRC cells, hnRNPA1 could regulate the transport of miR-106a-5p into exosomes by binding to a specific sequence (CAGGUA).

### Induction of M2 polarization by exosomal miR-106a-5p

**Fig. 4:**
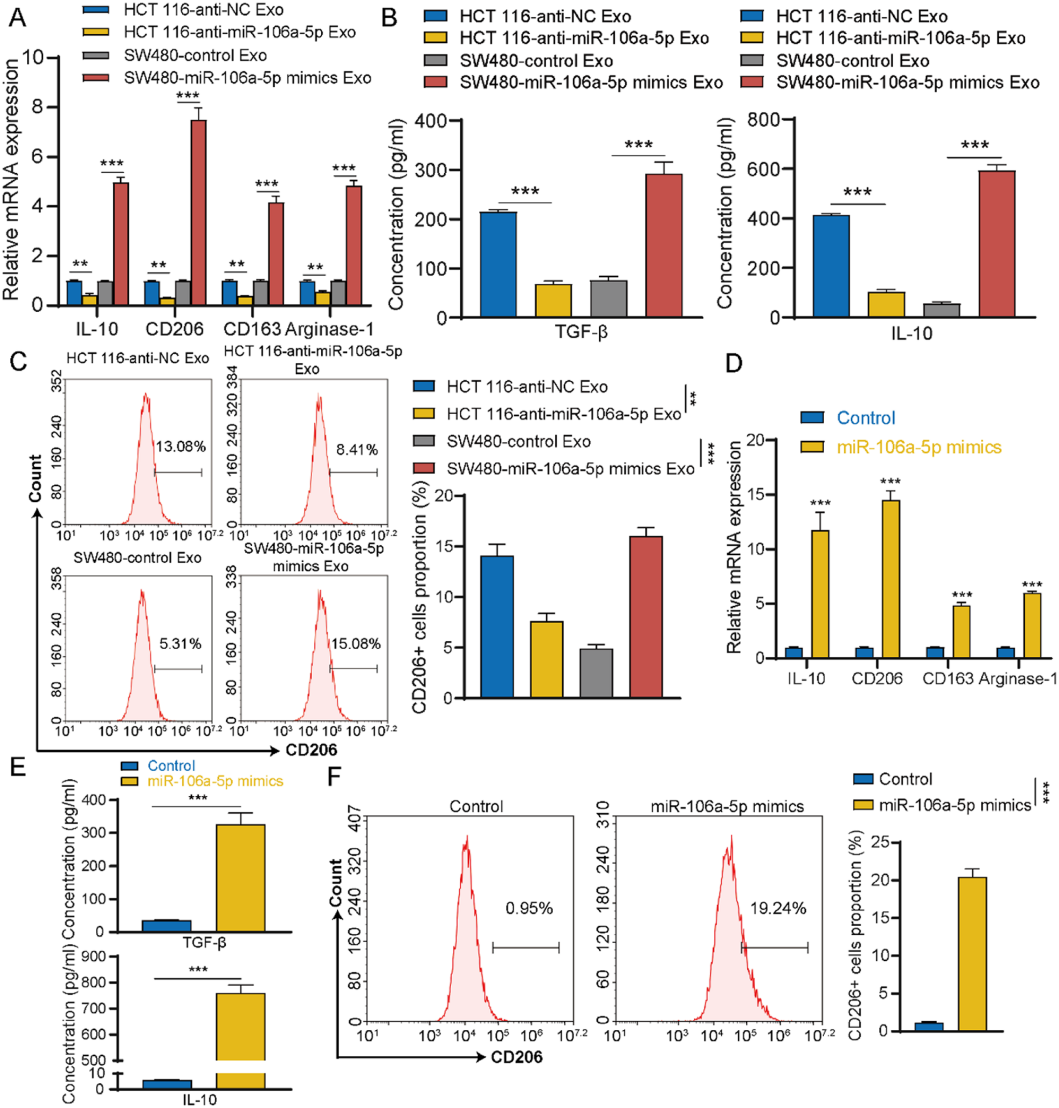
Induction of M2 polarization by exosomal miR-106a-5p. **(A)** qRT-PCR was used to detect changes in the expression of M2 (IL-10, CD206, CD163, and Arginase-1) macrophage marker genes in Mφ after incubated with different exosomes. **(B)** ELISA was used to assess the secretion of TGF-β and IL-10 by Mφ treated with different exosomes. **(C)** Flow cytometry was used to detect the proportion of CD206^+^ macrophages in Mφ after incubated with different exosomes. **(D)** qRT-PCR was employed to detect changes in the expression of M2 (IL-10, CD206, CD163, and Arginase-1) macrophage marker genes in Mφ following transfection with miR-106a-5p mimics. **(E)** ELISA was used to assess the secretion of TGF-β and IL-10 by Mφ transfected with miR-106a-5p mimics. **(F)** Flow cytometry was used to detect the proportion of CD206^+^ macrophages in Mφ after transfected with miR-106a-5p mimics. The data presented herein represent the outcomes of a minimum of three independent experiments and are depicted as the mean ± standard deviation (SD). ** *P* < 0.01, *** *P* < 0.001

After determining that macrophages could uptake exsomal miR-106a-5p, we further investigated whether miR-106a-5p could induce macrophages M2 polarization. Initially, miR-106a-5p was knocked down in HCT 116 cells and overexpressed in SW480 cells (named as HCT 116-anti-NC, HCT 116-anti-miR-106a-5p, SW480-control, SW480-miR-106a-5p mimics; Figure S4A-S4D). Then, exosomes were isolated and added to the Mφ. The results showed that knockdown of miR-106a-5p significantly weakened the ability of exosomes from HCT 116 cells to induce Mφ M2 polarization, while overexpression of miR-106a-5p significantly enhanced the ability of exosomes from SW480 cells to induce Mφ M2 polarization (Fig. 4A and C). Additionally, overexpression of miR-106a-5p in Mφ (Figure S4E) demonstrated that miR-106a-5p could significantly promote Mφ M2 polarization (Fig. 4D and F). These results suggest that exosomal miR-106a-5p derived from CRC cells could promote macrophages M2 polarization.

### Direct targeting of SOCS6 and activation of JAK2/STAT3 signaling pathway by exosomal miR-106a-5p in macrophages

**Fig. 5:**
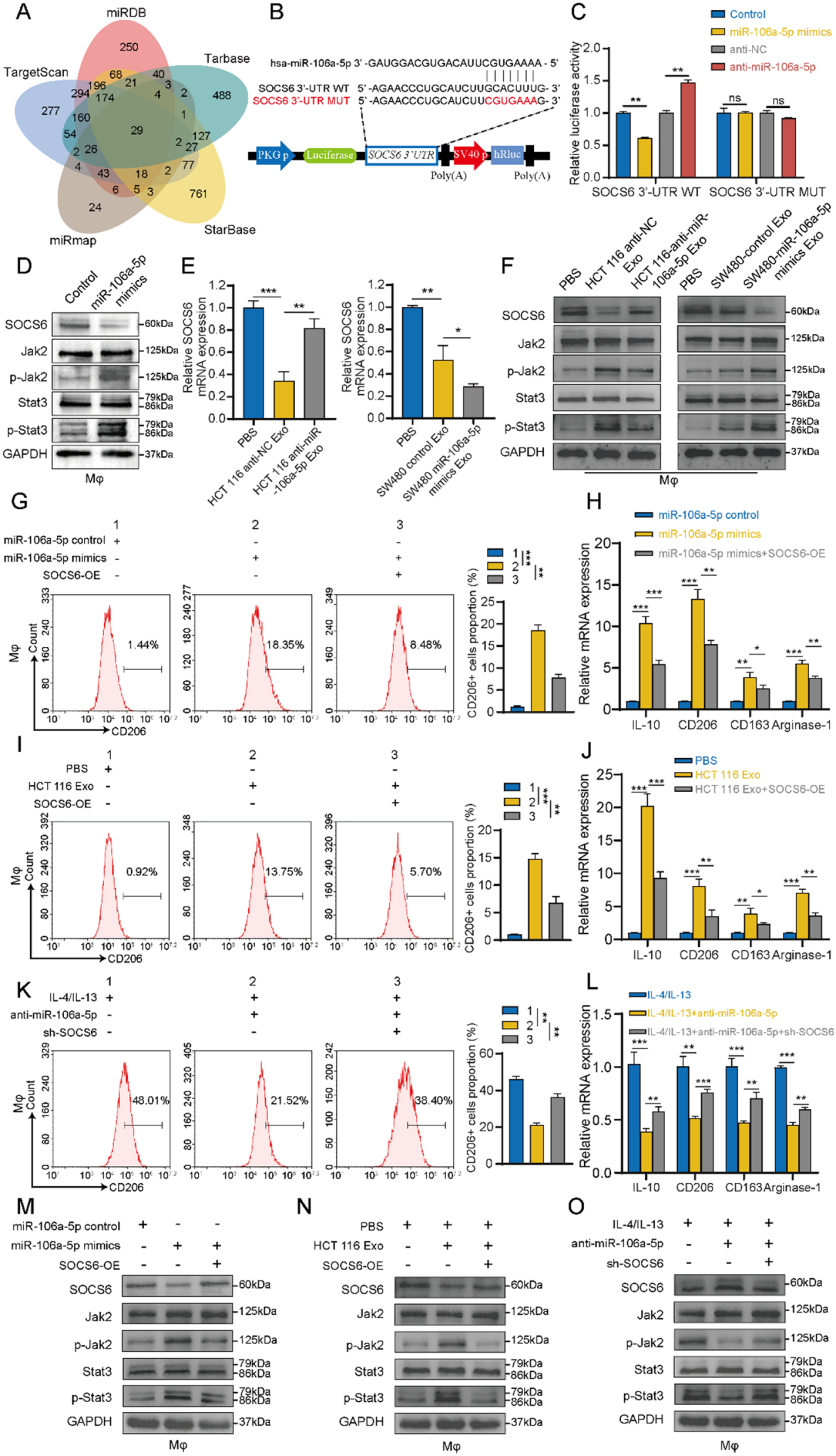
Direct targeting of SOCS6 and activation of JAK2/STAT3 signaling pathway by exosomal miR-106a-5p in macrophages. **A.** Venn plot showing the potential target genes predicted to bind with miR-106a-5p by five bioinformatics tools. **B.** The predicted binding sites of miR-106a-5p on the 3’UTR of SOCS6. **C.** In HEK 293T cells, dual-luciferase reporter gene assays were conducted using transfection with either wild or mutant SOCS6 3’-UTR plasma, along with miR-106a-5p mimics or inhibitor. The luciferase activity was detected 48 h post-transfection and normalized based on the ratio of firefly to renilla luciferase signals. **D.** 48 h following the transfection of miR-106a-5p mimics in Mφ, western blot was conducted to detect the expression of SOCS6 and the activation of the JAK2/STAT3 pathway. **E.** qRT-PCR was employed to detect the expression of SOCS6 in Mφ after treatment with different exosomes. **F.** 48 h following the co-culture of different exosomes in Mφ, western blot was conducted to detect the expression of SOCS6 and the activation of the JAK2/STAT3 pathway. **G-H.** Mφ were transfected with miR-106a-5p mimics alone or combined with SOCS6-OE. Flow cytometry was employed to assess the proportion of CD206^+^ macrophages (**G**). qRT-PCR was used to measure the expression of IL-10, CD206, CD163, and Arginase-1 (**H**). **I-J.** Mφ were treated with HCT 116 exosomes alone or concurrently transfected with SOCS6-OE. Flow cytometry was employed to determine the proportion of CD206^+^ macrophages (**I**). qRT-PCR was used to evaluate the expression of IL-10, CD206, CD163, and Arginase-1 (**J**). **K-L.** Mφ were differentiated into M2 macrophage using IL-4 (50 ng/mL) and IL-13 (50 ng/mL), followed by transfection with either miR-106a-5p inhibitor alone or combined with sh-SOCS6. Flow cytometry was utilized to assess the proportion of CD206^+^ macrophages (**K**). qRT-PCR was conducted to measure the expression of IL-10, CD206, CD163, and Arginase-1 (**L**). **M.** Mφ were transfected with miR-106a-5p mimics alone or combined with SOCS6-OE, followed by western blotting to detect SOCS6 expression and JAK2/STAT3 pathway activation. **N.** Mφ were treated with HCT 116 exosomes alone or concurrently transfected with SOCS6-OE, followed by western blotting to detect SOCS6 expression and JAK2/STAT3 pathway activation. **O.** Mφ were differentiated into M2 macrophages using IL-4 and IL-13, then transfected with miR-106a-5p inhibitor alone or combined with sh-SOCS6, followed by western blotting to detect SOCS6 expression and JAK2/STAT3 pathway activation. The data presented herein represent the outcomes of a minimum of three independent experiments and are depicted as the mean ± standard deviation (SD). ns = No significant difference, * *P* < 0.05, ** *P* < 0.01, *** *P* < 0.001

To elucidate the mechanism by which miR-106a-5p regulates the M2 polarization of macrophages, five bioinformatics tools (miRDB, Tarbase, StarBase, miRmap, and Targetscan) were employed to predict the target genes of miR-106a-5p (Fig. 5A). SOCS6 (suppressor of cytokine signaling 6), identified in the intersection of five databases, caught our attention. As a member of the suppressor of cytokine signaling (SOCS) family, SOCS6 could inhibit the activation of the JAK2/STAT3 signaling pathway [30]. Meanwhile, the activation of STAT3 is closely related to the macrophages M2 polarization [31]. Based on the predicted binding sites of miR-106a-5p on the 3’UTR of SOCS6 (Fig. 5B), dual-luciferase reporter gene assays were conducted. When HEK293T cells were co-transfected with Luc-SOCS6-3’UTR-WT plasmid and miR-106a-5p mimics, a significant decrease in luciferase activity was observed, while no change in luciferase activity was observed with co-transfection of Luc-SOCS6-3’UTR-MUT plasmid and miR-106a-5p mimics. Similarly, co-transfection of Luc-SOCS6-3’UTR-WT plasmid and miR-106a-5p inhibitor led to a marked increase in luciferase activity, but no change was observed with Luc-SOCS6-3’UTR-MUT plasmid and miR-106a-5p inhibitor (Fig. 5C). Western blot experiments further revealed that overexpression of miR-106a-5p in Mφ could downregulate SOCS6 and activate the JAK2/STAT3 signaling pathway (Fig. 5D). qRT-PCR showed that knockdown of miR-106a-5p significantly weakened the inhibitory effect of exosomes from HCT 116 cells on SOCS6 expression, while overexpression of miR-106a-5p significantly enhanced this inhibitory effect of exosomes from SW480 cells (Fig. 5E). Western blot also indicated that exosomes from HCT 116 and SW480 cells could significantly downregulate SOCS6 expression in Mφ, further activating the JAK2/STAT3 signaling pathway. This effect was significantly weakened with exosomes from HCT 116-anti-miR-106a-5p cells and significantly amplified with exosomes from SW480-miR-106a-5p-mimics cells (Fig. 5F).

Furthermore, flow cytometry showed that the increase in the proportion of CD206^+^ cells induced by miR-106a-5p mimics or exosomes from HCT 116 cells were attenuated when co-transfected with SOCS6 overexpression plasmid (SOCS6-OE) (Fig. 5G and I). qRT-PCR similarly indicated that overexpression of SOCS6 in Mφ weakened the upregulation of IL-10, CD206, CD163, and Arginase-1 induced by miR-106a-5p mimics or exosomes from HCT 116 cells (Fig. 5H and J). Additionally, flow cytometry demonstrated that the decrease in the proportion of CD206^+^ cells caused by miR-106a-5p inhibitor was reversed when co-transfected with an SOCS6 knockdown plasmid (sh-SOCS6) (Fig. 5K), and qRT-PCR showed that knockdown of SOCS6 in M2 macrophages restored the downregulation of IL-10, CD206, CD163, and Arginase-1 induced by miR-106a-5p inhibitor (Fig. 5L). Moreover, western blot further revealed that overexpression of SOCS6 in Mφ could reverse the downregulation of SOCS6 expression and activation of the JAK2/STAT3 signaling pathway caused by miR-106a-5p mimics or exosomes from HCT 116 cells (Fig. 5M and N). Knockdown of SOCS6 expression in M2 macrophages reversed the upregulation of SOCS6 expression and inhibition of the JAK2/STAT3 signaling pathway caused by the miR-106a-5p inhibitor (Fig. 5O). These results suggest that exosomal miR-106a-5p induces macrophages M2 polarization by directly targeting SOCS6, inhibiting its expression, and thereby activating the JAK2/STAT3 signaling pathway.

### Reciprocal promotion of CRC liver metastasis by exosomal miR-106a-5p-induced M2 macrophages

**Fig. 6:**
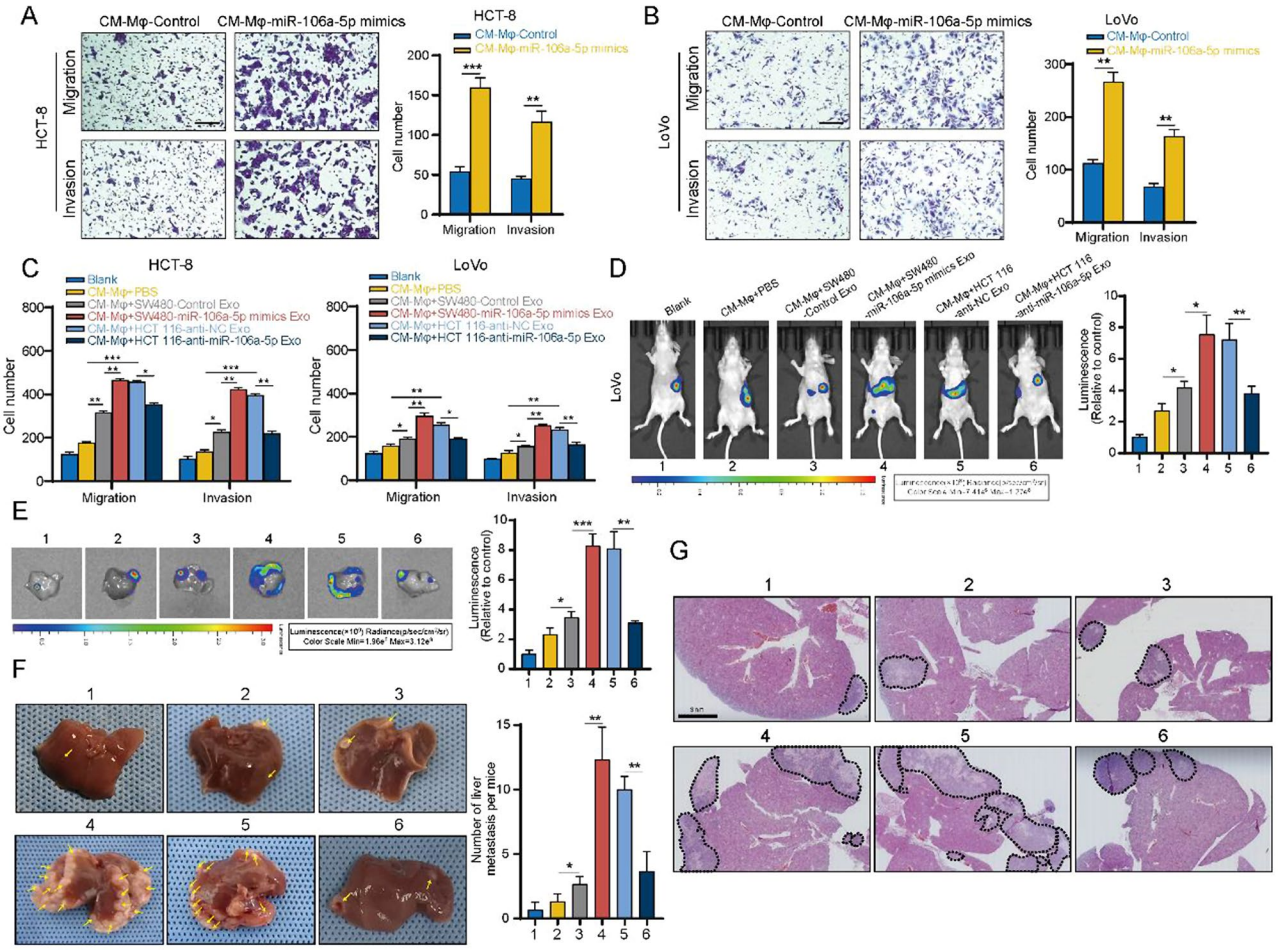
Reciprocal promotion of CRC liver metastasis by exosomal miR-106a-5p-induced M2 macrophages. A-B. Mφ cells were transfected with miR-106a-5p mimics or the corresponding control, and then the CM was added to the HCT-8 and LoVo cell lines. Transwell assays were performed to assess the migration and invasion capabilities of HCT-8 (**A**) and LoVo (**B**) cells treated with various CM. **C.** Mφ cells were co-cultured with exosomes derived from SW480-miR-106a-5p mimics, HCT 116-anti-miR-106a-5p, and their respective control groups. The co-cultured CM were then added to HCT-8 and LoVo cells. Transwell assays were performed to assess their migration and invasion capabilities. **D-G.** Representative bioluminescence images of liver metastasis in various treatment groups of nude mice (**D-E**). Representative photographs of liver metastasis (**F**) and HE staining (**G**) in various treatment groups of nude mice. The data presented herein represent the outcomes of a minimum of three independent experiments and are depicted as the mean ± standard deviation (SD). *P < 0.05, ** *P* < 0.01, *** *P* < 0.001

We have identified that exosomal miR-106a-5p can induce macrophages M2 polarization, and extensive research has shown that M2 macrophages can promote the malignant progression of tumors [9]. Consequently, we further explored whether exosomal miR-106a-5p induced M2 macrophages could in turn promote liver metastasis of CRC. We chose HCT-8 and LoVo cells for subsequent experiments. Initially, Mφ cells were transfected with miR-106a-5p mimics or the corresponding control, and then the conditioned medium (CM) was added to the HCT-8 and LoVo cell lines. Transwell showed that exogenous miR-106a-5p induced M2 macrophages significantly promoted migration and invasion of HCT-8 and LoVo cells (Fig. 6A and B).

Subsequently, Mφ cells were co-cultured with exosomes derived from SW480-miR-106a-5p mimics, HCT 116-anti-miR-106a-5p, and their respective control groups. The co-cultured CM were then added to HCT-8 and LoVo cells. In vitro, transwell demonstrated that M2 macrophages induced by exosomal miR-106a-5p from CRC cells (SW480-miR-106a-5p mimics, HCT 116-anti-miR-106a-5p, and their respective control groups) significantly enhanced the migration and invasion of HCT-8 and LoVo cells (Fig. 6C and S5C-S5D). In vivo experiments also showed that M2 macrophages induced by exosomal miR-106a-5p from CRC cells (SW480-miR-106a-5p mimics, HCT 116-anti-miR-106a-5p, and their respective control groups) significantly promoted liver metastasis of LoVo cells (Fig. 6D and G). These results indicated that M2 macrophages induced by exosomal miR-106a-5p could in turn promote liver metastasis of CRC.

### Elevated plasma exosomal miR-106a-5p as an independent prognostic marker in CRC

**Fig. 7:**
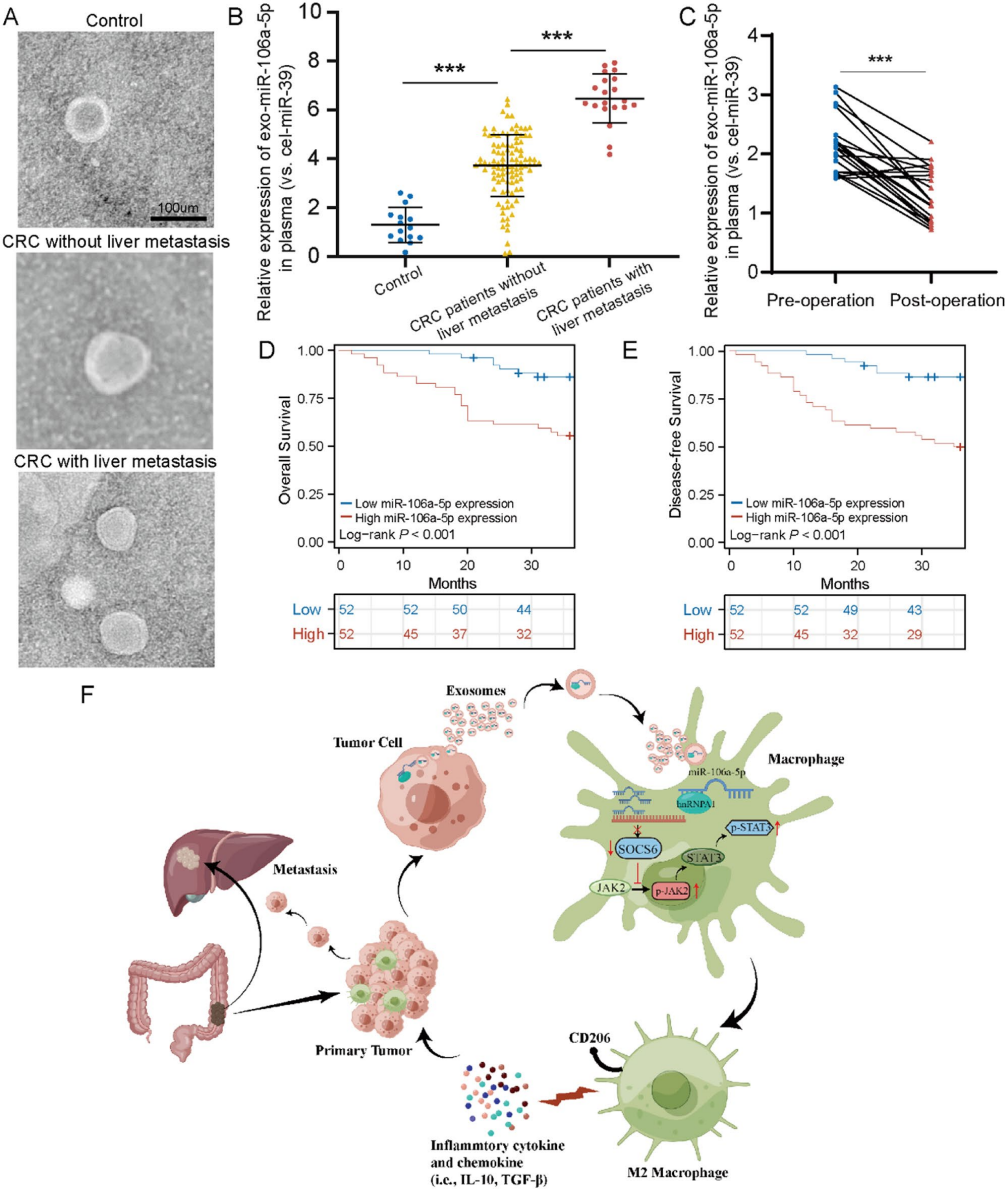
Elevated plasma exosomal miR-106a-5p as an independent prognostic marker in CRC. **(A)** TEM showed the typical structures of healthy individuals and CRC patients’ plasma exosomes. Scale bar = 100 μm. **(B)** qRT-PCR was used to detect the expression of miR-106a-5p in exosomes from healthy individuals, CRC patients without liver metastasis, and CRC patients with liver metastasis. **(C)** qRT-PCR was performed to assess the expression of miR-106a-5p in the plasma exosomes of 20 CRC patients both before and after undergoing surgery. **D-E.** High expression of exosomal miR-106a-5p correlated with shorter OS (**D**) and DFS (**E**). **F.** Schematic diagram depicting the positive feedback loop between highly metastatic CRC cells and M2 macrophages in CRC during liver metastasis. The data presented herein represent the outcomes of a minimum of three independent experiments and are depicted as the mean ± standard deviation (SD). *P < 0.05, ** *P* < 0.01, *** *P* < 0.001

To elucidate the clinical significance of exosomal miR-106a-5p in CRC patients, we isolated plasma exosomes from CRC patients and healthy individuals (Fig. 7A). qRT-PCR showed that plasma exosomal miR-106a-5p expression was elevated in CRC patients compared to healthy individuals. Moreover, the expression of miR-106a-5p in plasma exosomes was significantly higher in CRC patients with liver metastasis than in those without liver metastasis (Fig. 7B). Additionally, a dramatic decrease in the expression of plasma exosomal miR-106a-5p was observed in postoperative patients, suggesting that plasma exosomal miR-106a-5p is mainly produced by the tumor (Fig. 7C).

Furthermore, we found that increased expression levels of exosomal miR-106a-5p were closely associated with poor tumor differentiation, distant metastasis, and lymphatic/microvascular/perineural invasion (Table 1).

**Table 1:** Correlation of plasma exosomal miR-106a-5p level with clinicopathological factors in CRC patients (*n* = 104)

Variables	Patients	miR-106a-5p expression		χ^2^	*P* value
	(*n* = 104)	High (*n* = 52)	Low (*n* = 52)		
Gender				0.979	0.322
Male	59	27 (51.9)	32 (61.5)		
Female	45	25 (48.1)	20 (38.5)		
Age (years)				3.126	0.077
≤ 60	49	20 (38.5)	29 (55.8)		
> 60	55	32 (61.5)	23 (44.2)		
Tumor location				1.418	0.234
Colon	44	19 (36.5)	25 (48.1)		
Rectum	60	33 (63.5)	27 (51.9)		
Tumor diameter (cm)				2.719	0.099
≤ 5	68	30 (57.7)	38 (73.1)		
> 5	36	22 (42.3)	14 (26.9)		
Tumor differentiation				4.872	**0.027**
Poor	41	26 (50.0)	15 (28.8)		
Well / Moderate	63	26 (50.0)	37 (71.2)		
pT stage				1.846	0.174
T1-2	26	16 (30.8)	10 (19.2)		
T3-4	78	36 (69.2)	42 (80.8)		
pN stage				0.038	0.844
N0	51	26 (50.0)	25 (48.1)		
N1-2	53	26 (50.0)	27 (51.9)		
Distant metastasis				4.265	**0.039**
M0	79	35 (67.3)	44 (84.6)		
M1	25	17 (32.7)	8 (15.4)		
pTNM stage				0.639	0.424
I-II	42	19 (36.5)	23 (44.2)		
III-IV	62	33 (63.5)	29 (55.8)		
CEA (ng/ml)				2.556	0.110
≤ 5	62	27 (51.9)	35 (67.3)		
> 5	42	25 (48.1)	17 (32.7)		
CA19-9 (U/ml) ^#^				1.049	0.306
≤ 27	67	31 (59.6)	36 (69.2)		
> 27	37	21 (40.4)	16 (30.8)		
Lymphatic/microvascular/perineural invasion				7.278	**0.007**
Yes	35	24 (46.2)	11 (21.2)		
No	69	28 (53.8)	41 (78.8)		

* Defined by basic CEA or/AND CA19-9, CT or/AND MRI, or PET-CT

# The cut-off value is determined based on the diagnostic cut-off values utilized at our hospital

*P* values in bold indicate *P* < 0.05

Moreover, Cox proportional hazards regression analysis further revealed that plasma exosomal miR-106a-5p is an independent prognostic factor for CRC patients (Table 2). Concurrently, Kaplan-Meier survival analysis showed that patients with high expression of plasma exosomal miR-106a-5p had significantly lower OS and DFS than those with low expression (Fig. 7D and F). In summary, these findings suggest that plasma exosomal miR-106a-5p is highly expressed in CRC patients and associated with liver metastasis and poor prognosis. It could serve as a valuable independent prognostic factor for the diagnosis and treatment of CRC.

**Table 2:** Univariate and multivariate analysis of overall survival in CRC

Variables	Univariate analysis		Multivariate analysis	
	HR (95% CI)	*P* value	HR (95% CI)	*P* value
Gender (male vs. female)	0.86 (0.42–1.75)	0.671		
Age (> 60 years vs. ≤ 60 years)	1.66 (0.79–3.50)	0.180		
Tumor location (Rectum vs. Colon)	0.59 (0.29–1.20)	0.146		
Tumor diameter (> 5 cm vs. ≤ 5 cm)	2.86 (1.38–5.90)	**0.005**	1.75 (0.76–4.02)	0.186
Differentiation (well / moderate vs. poor)	0.21 (0.10–0.46)	**< 0.001**	0.28 (0.12–0.65)	**0.003**
pT stage (T3-4 vs. T1-2)	2.38 (0.83–6.81)	0.107		
pN stage (N1-2 vs. N0)	1.74 (0.83–3.66)	0.144		
Distant metastasis (M1 vs. M0)	5.88 (2.81–12.27)	**< 0.001**	3.24 (1.32–7.95)	**0.010**
pTNM stage (III - IV vs. I - II)	3.11 (1.27–7.62)	**0.013**	1.70 (0.57–5.07)	0.342
CEA (> 5 ng/ml vs. ≤ 5 ng/ml)	1.22 (0.59–2.50)	0.591		
CA19-9 (> 27 U/ml vs. ≤ 27 U/ml)	2.47 (1.20–5.06)	**0.014**	1.38 (0.60–3.14)	0.449
Lymphatic/microvascular/perineural invasion (Yes vs. No)	2.59 (1.26–5.31)	**0.010**	0.90 (0.39–2.11)	0.810
Exosomal miR-106a-5p expression (High vs. Low)	4.15 (1.78–9.68)	**0.001**	3.28 (1.35–7.96)	**0.009**

Abbreviations: HR, hazard ratio; CI, confidence interval

*P* values in bold indicate *P* < 0.05

## Discussion

The TME, often referred to as the “soil” of the tumor, is a dynamic and intricately regulated system that plays a pivotal role in tumor development and metastasis [32, 33]. Among the various components within the TME, macrophages hold a central position, and their phenotypic alterations are known to influence tumor metastasis significantly [34]. Generally, categorized as classically activated M1 macrophages with anti-tumor effects and alternatively activated M2 macrophages promoting tumor progression [35], the plasticity of macrophages within the TME has been widely explored.

Previous studies have showed that macrophages uptake exosomes from triple-negative breast cancer cells enriched with miR-184-3p, which induce M2 polarization and promote tumor metastasis via downregulating EGR1 to inhibit the JNK signaling pathway [36]. Through the analysis of single-cell RNA sequencing data, we discovered higher M2 macrophage infiltration in CRC tissues with liver metastasis, suggesting a potential role in the metastatic cascade. However, the detailed mechanism through which CRC cells regulate macrophages in this context remain elusive.

The classical understanding of tumor cells induce macrophage polarization via secretion of cytokines or chemokines has been expanded with increasing awareness of exosome-mediated intercellular interactions within the TME [37–39]. Exosomes, as carriers of bioactive molecules such as miRNA and proteins, play a crucial role in modulating macrophage polarization and tumor progression [40–42]. Our study delved into this aspect, revealing that exosomes from highly metastatic CRC cells induced greater M2 macrophage polarization than those from low metastatic CRC cells. Further analysis of exosomal miRNA sequencing data from the GEO database identified miR-106a-5p as enriched in exosomes from CRC patients with liver metastasis and highly metastatic CRC cells. Despite the well-established role of miR-106a-5p in promoting malignant progression in various cancers [43–45], the specific function of exosomal miR-106a-5p derived from CRC cells had not been clarified until our study. Our findings demonstrated that exosomal miR-106a-5p strongly induced M2 macrophage polarization, prompting further investigation into the mechanisms governing its transfer into exosomes.

Specific RBPs have been implicated in regulating miRNA packaging into exosomes [46]. Heterogeneous nuclear ribonucleoproteins (hnRNPs), a class of RBPs, play various biological functions beyond their initially identified role in pre-mRNA splicing [47]. Our study identified hnRNPA1 as a key player in regulating the packaging of miR-106a-5p into exosomes. This aligns with previous research demonstrating hnRNPA1’s involvement in mediating the transfer of miRNAs via exosomes in other cancer types [48]. Targeting hnRNPA1 provides a potential avenue for therapeutic interventions aimed at modulating the TME during CRC treatment.

Going further into the mechanistic insights, our study unveiled that exosomal miR-106a-5p induced M2 macrophage polarization by downregulating SOCS6 and activating the JAK2/STAT3 signaling pathway. SOCS6, a member of the suppressor of cytokine signaling family, is known for the role in inhibiting the JAK2/STAT3 signaling pathway [49]. While SOCS6 has been predominantly studied in tumor cells [50, 51], our research shed light on its role in immune cells, particularly macrophages. The identified regulatory axis suggests that inhibiting the phosphorylation of STAT3 could potentially alter the TME, suppressing tumor progression. Furthermore, the bidirectional interaction between tumor-derived exosomes and M2 macrophages was explored in the context of CRC liver metastasis, revealing that these M2 macrophages reciprocally promoted CRC liver metastasis.

Our clinical investigations into plasma exosomal miR-106a-5p levels in CRC patients provided translational relevance to our findings. Elevated expression of plasma exosomal miR-106a-5p was observed in CRC patients, particularly those with liver metastasis, and significantly decreased postoperatively, emphasizing the tumor as a primary source. Correlations were established between high exosomal miR-106a-5p expression and adverse clinicopathological features, including poor tumor differentiation, distant metastasis, and invasion characteristics. Furthermore, Cox proportional hazards regression analysis identified plasma exosomal miR-106a-5p as an independent prognostic factor for CRC patients. Kaplan-Meier survival analysis corroborated these findings, demonstrating lower OS and DFS in patients with high miR-106a-5p expression. This identifies plasma exosomal miR-106a-5p as a promising independent prognostic biomarker for CRC.

## Conclusion

In conclusion, our study provides comprehensive insights into the role of CRC-derived exosomal miR-106a-5p in mediating cell-to-cell crosstalk between CRC cells and macrophages. This newly identified regulatory axis offers potential therapeutic targets and presents plasma exosomal miR-106a-5p as a specific and valuable biomarker for CRC diagnosis and treatment. The bidirectional interaction between exosomal miR-106a-5p-induced M2 macrophages and CRC cells sheds light on the complex dynamics within the TME, particularly in the context of CRC liver metastasis. The identification of hnRNPA1 as a key regulator of miRNA packaging into exosomes further adds to the mechanistic understanding, providing avenues for the development of targeted therapeutic strategies aimed at modulating the TME during CRC treatment.

## Electronic supplementary material

Below is the link to the electronic supplementary material.

Supplementary Material 1

Supplementary Material 2

Supplementary Material 3

Supplementary Material 4

Supplementary Material 5

Supplementary Material 6: Fig. S1 Single-cell expression atlas of CRC with or without liver metastasis. (A) Uniform manifold approximation and projection (UMAP) plot of the major cell types in all samples (n = 13). (B) Dot plot exhibiting the marker genes across the cell types. (C) Histogram showed the proportions of cell types in CRC tissues with or without liver metastasis. (D) UMAP plot of myeloid clusters. (E) Histogram showed the proportions of myeloid types in CRC tissues with or without liver metastasis. (F) Heatmap exhibiting the marker genes across macrophage subsets. (G) Histogram showed the proportions of macrophage types in CRC tissues with or without liver metastasis, Fig. S2 Transwell assay was conducted to explore the metastatic capability of four different CRC cells. ** p < 0.01, *** p < 0.001, Fig. S3 (A) Western blot was employed to assess hnRNPA1 expression in samples obtained from miRNA pulldowns, utilizing nuclear, cytoplasmic, or exosomal lysates from SW620 cells. (B) RIP assays were conducted using an anti-hnRNPA1 antibody (or IgG as a control) on lysates derived from SW620 cells or exosomes. qRT-PCR was employed to quantify miR-106a-5p levels in the immunoprecipitated samples, expressed as percentages relative to the input (% input). *** p < 0.001, Fig. S4 (A) qRT-PCR was performed to confirm the efficiency of lentivirus-mediated knockdown of miR-106a-5p in HCT 116 cells. (B) qRT-PCR was performed to detect the expression of miR-106a-5p in the exosomes of HCT 116 cells treated with miR-106a-5p knockdown lentivirus and lentiviral control. (C) qRT-PCR was performed to confirm the efficiency of lentivirus-mediated overexpression of miR-106a-5p in SW480 cells. (D) qRT-PCR was performed to detect the expression of miR-106a-5p in the exosomes of SW480 cells treated with miR-106a-5p overexpression lentivirus and lentiviral control. E-F qRT-PCR was performed to detect the expression of miR-106a-5p in Mφ cells after transfection with miR-106a-5p mimics (E) and in M2 macrophages after transfection with miR-106a-5p inhibitor (F). ** p < 0.01, *** p < 0.001, Fig. S5 A-B Western blot was performed to detect the expression of SOCS6 in M2 macrophages after transfection with sh-SOCS6 (A) and in Mφ cells after transfection with SOCS6-OE (B). C-D Transwell assays were performed to assess the migration and invasion capabilities of HCT-8 (C) and LoVo (D) cells treated with various CM

## Data Availability

Original data in our study are available upon request.

## Abbreviations

TME: Tumor microenvironment
CRC: Colorectal cancer
SOCS6: Suppressor of cytokine signaling 6
JAK2: Janus kinase 2
STAT3: Signal transducer and activator of transcription 3
hnRNPA1: Heterogeneous nuclear ribonucleoprotein A1
miRNA: MicroRNA
OS: Overall survival
DFS: Disease-free survival
TAMs: Tumor-associated macrophages
MRC1: Mannose receptor C-type 1
qRT-PCR: Quantitative real-time polymerase chain reaction
PMA: Phorbol 12-myristate 13-acetate
ELISA: Enzyme linked immunosorbent assay
RBPs: RNA-binding proteins
RIP: RNA-binding protein immunoprecipitation
CM: Conditioned medium
