# Supplementary material for: Exosomal miR-106a-5p from highly metastatic colorectal cancer cells drives liver metastasis by inducing macrophage M2 polarization in the tumor microenvironment
Source: J Exp Clin Cancer Res. 2024 Oct 9;43:281. doi: 10.1186/s13046-024-03204-7 (PMC11462797; doi:10.1186/s13046-024-03204-7)
Supplement: Supplementary file 1 — Supplementary Material 1 [file 13046_2024_3204_MOESM1_ESM.docx]

**Table S1. Primers and Oligonucleotides sequences**

| **Variable** | **Sequence (5'-3')** |
| --- | --- |
| Primers |  |
| IL-10 Forward | TCAAGGCGCATGTGAACTCC |
| IL-10 Reverse | GATGTCAAACTCACTCATGGCT |
| CD206 Forward | GGGTTGCTATCACTCTCTATGC |
| CD206 Reverse | TTTCTTGTCTGTTGCCGTAGTT |
| CD163 Forward | TTTGTCAACTTGAGTCCCTTCAC |
| CD163 Reverse | TCCCGCTACACTTGTTTTCAC |
| Arginase-1 Forward | GTGGAAACTTGCATGGACAAC |
| Arginase-1 Reverse | AATCCTGGCACATCGGGAATC |
| IL-1β Forward | ATGATGGCTTATTACAGTGGCAA |
| IL-1β Reverse | GTCGGAGATTCGTAGCTGGA |
| iNOS Forward | TTCAGTATCACAACCTCAGCAAG |
| iNOS Reverse | TGGACCTGCAAGTTAAAATCCC |
| CD68 Forward | CTTCTCTCATTCCCCTATGGACA |
| CD68 Reverse | GAAGGACACATTGTACTCCACC |
| hnRNPA1 Forward | AAGCCCTGTCAAAGCAAGAGATGG |
| hnRNPA1 Reverse | CCACCACGACCACTGAAGTTTCC |
| SOCS6 Forward | TCTCTGCGGTTCCTCCTCAAGTG |
| SOCS6 Reverse | CAATCAACAAGCCATTCACGGACTG |
| GAPDH Forward | GGAGCGAGATCCCTCCAAAAT |
| GAPDH Reverse | GGCTGTTGTCATACTTCTCATGG |
| miR-106a-5p Forward | CGGCAAAAGTGCTTACAGTGC |
| miR-17-5p Forward | CGGCCAAAGTGCTTACAGTGC |
| miR-423-5p Forward | AGCTGAGGGGCAGAGAGCGA |
| miR-140-3p Forward | GCGGCCTACCACAGGGTAGAA |
| cel-miR-39-3p Forward | GGGCGACTCACCGGGTGTAAAT |
| U6 Forward | CCTGCTTCGGCAGCACA |
| Oligonucleotides |  |
| miR-106a-5p mimics | AAAAGUGCUUACAGUGCAGGUAG |
| miR-106a-5p inhibitor | CUACCUGCACUGUAAGCACUUUU |
| cel-miR-39-3p mimics | UCACCGGGUGUAAAUCAGCUUG |
| sh-hnRNPA1-1 | GACCCATGAAGGGAGGAAATTT |
| sh-hnRNPA1-2 | GGCTTTGGGTTTGTCACATAT |
| sh-NC | CCTAAGGTTAAGTCGCCCTCG |
| sh-SOCS6 | AGAUGGAGGUGAGAGUCAAGG |
