# Supplementary material for: Exosomal miR-106a-5p from highly metastatic colorectal cancer cells drives liver metastasis by inducing macrophage M2 polarization in the tumor microenvironment
Source: J Exp Clin Cancer Res. 2024 Oct 9;43:281. doi: 10.1186/s13046-024-03204-7 (PMC11462797; doi:10.1186/s13046-024-03204-7)
Supplement: Supplementary file 2 — Supplementary Material 2 [file 13046_2024_3204_MOESM2_ESM.docx]

**Table S2. Antibodies used in the present study**

| **Product** | **Concentration** | **Clone Number** | **Company** |
| --- | --- | --- | --- |
| **Western blot** |  |  |  |
| Primary antibody |  |  |  |
| CD9 | 1:500 | sc-13118 | Santa Cruz |
| TSG101 | 1:500 | sc-7964 | Santa Cruz |
| Alix | 1:500 | sc-53540 | Santa Cruz |
| Calnexin | 1:500 | sc-23954 | Santa Cruz |
| hnRNPA1 | 1:1000 | #8443 | Cell Signaling Technology |
| Histone H3 | 1:2000 | #4499 | Cell Signaling Technology |
| β-actin | 1:1000 | #8457 | Cell Signaling Technology |
| SOCS6 | 1:1000 | A9957 | ABclonal |
| Jak2 | 1:1000 | #3230 | Cell Signaling Technology |
| p-Jak2 | 1:1000 | #3771 | Cell Signaling Technology |
| Stat3 | 1:1000 | #9139 | Cell Signaling Technology |
| p-Stat3 | 1:2000 | #9145 | Cell Signaling Technology |
| GAPDH | 1:50000 | 60004-1-Ig | Proteintech |
| Secondary antibody |  |  |  |
| Anti-rabbit IgG-HRP | 1:10000 | SA00001-2 | Proteintech |
| Anti-mouse IgG-HRP | 1:10000 | SA00001-1 | Proteintech |
| **IF and FCM** |  |  |  |
| Primary antibody |  |  |  |
| CD206 |  | 18704-1-AP | Proteintech |
| CD68 |  | 66231-2-Ig | Proteintech |
| Secondary antibody |  |  |  |
| Anti-Rabbit IgG H&L (Alexa Fluor® 488) |  | ab150077 | Abcam |
