# Supplementary material for: Exosomal miR-106a-5p from highly metastatic colorectal cancer cells drives liver metastasis by inducing macrophage M2 polarization in the tumor microenvironment
Source: J Exp Clin Cancer Res. 2024 Oct 9;43:281. doi: 10.1186/s13046-024-03204-7 (PMC11462797; doi:10.1186/s13046-024-03204-7)
Supplement: Supplementary file 3 — Supplementary Material 3 [file 13046_2024_3204_MOESM3_ESM.docx]

**Table S3. Gene ontology of the seven candidate RBPs**

| **Gene ontology** | **GO ID** | **GO term** |
| --- | --- | --- |
| ***FUS*** | | |
| Biological process | GO:0006355 | regulation of transcription, DNA-templated |
| Biological process | GO:0008380 | RNA splicing |
| Biological process | GO:1903508 | positive regulation of nucleic acid-templated transcription |
| Biological process | GO:0051260 | protein homooligomerization |
| Biological process | GO:0006357 | regulation of transcription by RNA polymerase II |
| Biological process | GO:0043484 | regulation of RNA splicing |
| Biological process | GO:0000398 | mRNA splicing, via spliceosome |
| Biological process | GO:1905168 | positive regulation of double-strand break repair via homologous recombination |
| Biological process | GO:0010467 | gene expression |
| Biological process | GO:0048255 | mRNA stabilization |
| Biological process | GO:0071277 | cellular response to calcium ion |
| Biological process | GO:0000398 | mRNA splicing, via spliceosome |
| Biological process | GO:1903506 | regulation of nucleic acid-templated transcription |
| Cellular component | GO:0005634 | nucleus |
| Cellular component | GO:0005654 | nucleoplasm |
| Cellular component | GO:0005737 | cytoplasm |
| Cellular component | GO:0005844 | polysome |
| Cellular component | GO:0030425 | dendrite |
| Cellular component | GO:0043025 | neuronal cell body |
| Cellular component | GO:0043197 | dendritic spine |
| Cellular component | GO:0043204 | perikaryon |
| Cellular component | GO:0044327 | dendritic spine head |
| Cellular component | GO:0048471 | perinuclear region of cytoplasm |
| Molecular function | GO:0003723 | RNA binding |
| Molecular function | GO:0003676 | nucleic acid binding |
| Molecular function | GO:0046872 | metal ion binding |
| Molecular function | GO:0003677 | DNA binding |
| Molecular function | GO:0005515 | protein binding |
| Molecular function | GO:0042802 | identical protein binding |
| Molecular function | GO:0003712 | transcription coregulator activity |
| Molecular function | GO:0003682 | chromatin binding |
| Molecular function | GO:0003713 | transcription coactivator activity |
| Molecular function | GO:0003730 | mRNA 3'-UTR binding |
| Molecular function | GO:0030331 | estrogen receptor binding |
| Molecular function | GO:0031489 | myosin V binding |
| Molecular function | GO:0035255 | ionotropic glutamate receptor binding |
| Molecular function | GO:0046965 | retinoid X receptor binding |
| Molecular function | GO:0046966 | thyroid hormone receptor binding |
| Molecular function | GO:0000166 | nucleotide binding |
| Molecular function | GO:0008270 | zinc ion binding |
| Molecular function | GO:0044822 | poly(A) RNA binding |
| ***YTHDC1*** | | |
| Biological process | GO:0006376 | mRNA splice site selection |
| Biological process | GO:0048024 | regulation of mRNA splicing, via spliceosome |
| Cellular component | GO:0005634 | nucleus |
| Cellular component | GO:0016607 | nuclear speck |
| Molecular function | GO:0003723 | RNA binding |
| Molecular function | GO:0005515 | protein binding |
| Molecular function | GO:0044822 | poly(A) RNA binding |
| Molecular function | GO:1990247 | N6-methyladenosine-containing RNA binding |
| ***NONO*** | | |
| Biological process | GO:0000398 | mRNA splicing, via spliceosome |
| Biological process | GO:0006281 | DNA repair |
| Biological process | GO:0006310 | DNA recombination |
| Biological process | GO:0006351 | Transcription, DNA-templated |
| Biological process | GO:0006355 | regulation of transcription, DNA-templated |
| Biological process | GO:0006397 | mRNA processing |
| Biological process | GO:0007623 | circadian rhythm |
| Biological process | GO:0008380 | RNA splicing |
| Biological process | GO:0042752 | regulation of circadian rhythm |
| Biological process | GO:0045892 | negative regulation of transcription, DNA-templated |
| Biological process | GO:1903377 | negative regulation of oxidative stress-induced neuron intrinsic apoptotic signaling pathway |
| Cellular component | GO:0005634 | nucleus |
| Cellular component | GO:0005654 | nucleoplasm |
| Cellular component | GO:0005730 | nucleolus |
| Cellular component | GO:0016020 | membrane |
| Cellular component | GO:0016363 | nuclear matrix |
| Cellular component | GO:0016607 | nuclear speck |
| Cellular component | GO:0042382 | paraspeckles |
| Cellular component | GO:0090575 | RNA polymerase II transcription factor complex |
| Molecular function | GO:0000166 | nucleotide binding |
| Molecular function | GO:0000976 | transcription regulatory region sequence-specific DNA binding |
| Molecular function | GO:0000980 | RNA polymerase II distal enhancer sequence-specific DNA binding |
| Molecular function | GO:0001047 | core promoter binding |
| Molecular function | GO:0003682 | chromatin binding |
| Molecular function | GO:0005515 | protein binding |
| Molecular function | GO:0042802 | identical protein binding |
| Molecular function | GO:0044822 | poly(A) RNA binding |
| ***ELAVL1*** | | |
| Biological process | GO:0000398 | mRNA splicing, via spliceosome |
| Biological process | GO:0007275 | multicellular organism development |
| Biological process | GO:0043488 | regulation of mRNA stability |
| Biological process | GO:0045727 | positive regulation of translation |
| Biological process | GO:0048255 | mRNA stabilization |
| Biological process | GO:0070935 | 3'-UTR-mediated mRNA stabilization |
| Biological process | GO:2000036 | regulation of stem cell population maintenance |
| Cellular component | GO:0005634 | nucleus |
| Cellular component | GO:0005654 | nucleoplasm |
| Cellular component | GO:0005737 | cytoplasm |
| Cellular component | GO:0005829 | cytosol |
| Cellular component | GO:0016020 | membrane |
| Molecular function | GO:0000166 | nucleotide binding |
| Molecular function | GO:0003723 | RNA binding |
| Molecular function | GO:0003725 | double-stranded RNA binding |
| Molecular function | GO:0003729 | mRNA binding |
| Molecular function | GO:0003730 | mRNA 3'-UTR binding |
| Molecular function | GO:0005515 | protein binding |
| Molecular function | GO:0017091 | AU-rich element binding |
| Molecular function | GO:0019901 | protein kinase binding |
| Molecular function | GO:0035925 | mRNA 3'-UTR AU-rich region binding |
| Molecular function | GO:0042803 | protein homodimerization activity |
| Molecular function | GO:0044822 | poly(A) RNA binding |
| ***HNRNPA1*** | | |
| Biological process | GO:0000398 | mRNA splicing, via spliceosome |
| Biological process | GO:0006397 | mRNA processing |
| Biological process | GO:0006405 | RNA export from nucleus |
| Biological process | GO:0008543 | fibroblast growth factor receptor signaling pathway |
| Biological process | GO:0010467 | gene expression |
| Biological process | GO:0016032 | viral process |
| Biological process | GO:0032211 | negative regulation of telomere maintenance via telomerase |
| Biological process | GO:0032212 | positive regulation of telomere maintenance via telomerase |
| Biological process | GO:0051028 | mRNA transport |
| Biological process | GO:0051168 | nuclear export |
| Biological process | GO:0051170 | nuclear import |
| Cellular component | GO:0005634 | nucleus |
| Cellular component | GO:0005654 | nucleoplasm |
| Cellular component | GO:0005681 | spliceosomal complex |
| Cellular component | GO:0005737 | cytoplasm |
| Cellular component | GO:0016020 | membrane |
| Cellular component | GO:0030529 | intracellular ribonucleoprotein complex |
| Cellular component | GO:0070062 | extracellular exosome |
| Cellular component | GO:0071013 | catalytic step 2 spliceosome |
| Molecular function | GO:0000166 | nucleotide binding |
| Molecular function | GO:0003697 | single-stranded DNA binding |
| Molecular function | GO:0003723 | RNA binding |
| Molecular function | GO:0003727 | single-stranded RNA binding |
| Molecular function | GO:0005515 | protein binding |
| Molecular function | GO:0044822 | poly(A) RNA binding |
| Molecular function | GO:0061752 | telomeric repeat-containing RNA binding |
| Molecular function | GO:0098505 | G-rich strand telomeric DNA binding |
| ***ZRANB2*** | | |
| Biological process | GO:0006355 | regulation of transcription, DNA-templated |
| Biological process | GO:0006397 | mRNA processing |
| Biological process | GO:0008380 | RNA splicing |
| Cellular component | GO:0005634 | nucleus |
| Cellular component | GO:0005654 | nucleoplasm |
| Molecular function | GO:0003700 | transcription factor activity, sequence-specific DNA binding |
| Molecular function | GO:0003723 | RNA binding |
| Molecular function | GO:0005515 | protein binding |
| Molecular function | GO:0008270 | zinc ion binding |
| Molecular function | GO:0044822 | poly(A) RNA binding |
| ***KHSRP*** | | |
| Biological process | GO:0000375 | RNA splicing, via transesterification reactions |
| Biological process | GO:0006351 | transcription, DNA-templated |
| Biological process | GO:0006355 | regulation of transcription, DNA-templated |
| Biological process | GO:0006397 | mRNA processing |
| Biological process | GO:0006402 | mRNA catabolic process |
| Biological process | GO:0008380 | RNA splicing |
| Biological process | GO:0043488 | regulation of mRNA stability |
| Biological process | GO:0051028 | mRNA transport |
| Biological process | GO:2000628 | regulation of miRNA metabolic process |
| Cellular component | GO:0005654 | nucleoplasm |
| Cellular component | GO:0005829 | cytosol |
| Cellular component | GO:0010494 | cytoplasmic stress granule |
| Cellular component | GO:0016020 | membrane |
| Cellular component | GO:0030425 | dendrite |
| Cellular component | GO:0043025 | neuronal cell body |
| Molecular function | GO:0003677 | DNA binding |
| Molecular function | GO:0003730 | mRNA 3'-UTR binding |
| Molecular function | GO:0044822 | poly(A) RNA binding |
