# Supplementary material for: Exosomal miR-106a-5p from highly metastatic colorectal cancer cells drives liver metastasis by inducing macrophage M2 polarization in the tumor microenvironment
Source: J Exp Clin Cancer Res. 2024 Oct 9;43:281. doi: 10.1186/s13046-024-03204-7 (PMC11462797; doi:10.1186/s13046-024-03204-7)
Supplement: Supplementary file 4 — Supplementary Material 4 [file 13046_2024_3204_MOESM4_ESM.docx]

**Table S4. The GEO samples ID included in the present study**

| **GSE205506** | **GSE178318** |
| --- | --- |
| GSM6213970 | GSM5387660 |
| GSM6213971 | GSM5387662 |
| GSM6213973 | GSM5387667 |
| GSM6213976 |  |
| GSM6213980 |  |
| GSM6213983 |  |
| GSM6213988 |  |
| GSM6213991 |  |
| GSM6213994 |  |
| GSM6213995 |  |
