# Supplementary material for: Exosomal miR-106a-5p from highly metastatic colorectal cancer cells drives liver metastasis by inducing macrophage M2 polarization in the tumor microenvironment
Source: J Exp Clin Cancer Res. 2024 Oct 9;43:281. doi: 10.1186/s13046-024-03204-7 (PMC11462797; doi:10.1186/s13046-024-03204-7)
Supplement: Supplementary file 5 — Supplementary Material 5 [file 13046_2024_3204_MOESM5_ESM.docx]

**Supplementary Materials and Methods**

**Single-cell RNA sequencing analysis**

We analyzed the single-cell RNA sequencing data, which obtained from GEO database (GSE205506, GSE178318). The detail GEO samples ID was exhibited in the Table S4. Quality control and gene expression matrices were performed by R software (Version 4.0.5) with Seurat v4 package[1]. Cells meeting the following criteria were defined as low-quality cells and were removed: (1) < 200 or > 5000 genes, (2) > 50000 unique molecular identiﬁers (UMIs), (3) > 25% mitochondrial genes proportion. Then, the expression matrix underwent normalization using the ‘NormalizeData’ function. Subsequently, the ‘FindVariableFeatures’ function was utilized to identify 2000 genes with significant variability across cells. For multi-sample integration, the ‘FindIntegrationAnchors’ function was utilized to find anchors among individual samples. The batch-corrected expression matrix was obtained by the ‘IntegrateData’ function. Utilizing this batch-corrected expression matrix, a Principal Component Analysis (PCA) on the top 2000 variable features was performed by using ‘RunPCA’ function. The ‘FindNeighbors’ and ‘FindClusters’ functions were used to cell cluster analysis with the first 50 principal components (PCs). UMAP was executed on the top 50 PCs through the ‘RunUMAP’ function. Then, the ‘FindAllMarkers’ function was utilized to identify marker genes for each cell clusters. Finally, these clusters were annotated by the known markers. For the subclustering of major cell types, we first extracted these cells from the overview integrated dataset. Subsequently, the expression matrix for these cells was processed as above described, involving integration, scaling, PCA, and clustering.

**Small RNA sequencing analysis**

The miRNA sequencing data was obtained from the GEO database (GSE115114, GSE123708). Differential miRNA expression analysis was executing using limma package. miRNAs with a *P*-value less than 0.05 and a |logFC| greater than 1 were considered to be differentially expressed.

**Cell culture and transfection**

The normal colon mucosal cell line NCM460, HEK293T, THP-1 and CRC cell lines were provided by American Type Culture Collection. All cells underwent STR (Short Tandem Repeat) identification. NCM460, HCT-8, DLD-1 and THP-1 were cultured with RPMI-1640. HEK293T, SW620, Caco-2 and HCT 116 were cultured with Dulbecco's Modified Eagle Medium (DMEM). SW480 and LoVo were cultured with L15 and F12K, respectively. All medium was added with 10% fetal bovine serum (FBS) (Avantor, PA, USA) and 1% penicillin-streptomycin (Solarbio, Beijing, China). During the experimental period, regular testing for mycoplasma was performed on the cells, with no mycoplasma contamination detected. All transient transfections were executed using Lipofectamine 2000 based on the manufacturer’s instructions. (Invitrogen, Carlsbad, CA, USA). The lentiviral transfection was executed according to the manufacturer’s instructions (Genechem, Shanghai, China). All the oligonucleotide sequences are exhibited in Table S1.

**Immunofluorescence and hematoxylin-eosin (HE) staining**

For tissue immunofluorescence and HE, the tissue was first subjected to fixation, dehydration, paraffin embedding and sectioning. Then, the tissue slices were baked at 65℃ for 2 h and deparaffinized with xylene and ethanol. Antigen retrieval was performed using EDTA antigen retrieval solution (pH 9.0). After blocking with goat serum for 2 h, tissue slices were incubated with primary antibody for overnight at 4℃. Then, tissue slices were incubated in the dark at room temperature with fluorescently labeled secondary antibody for 2 h before the application of DAPI for nuclear staining. Image acquisition was executed using a fluorescence microscope (Zeiss, Germany). For cell immunofluorescence, the cells were first treated with 4% paraformaldehyde and Triton X-100, then blocking with goat serum. Subsequently, the cells were sequentially incubated with the primary antibody and fluorescently labeled secondary antibody. For tissue HE staining, we followed the manufacturer's instructions (G1120) (Solarbio, Beijing, China). The antibodies are listed in Table S2.

**Exosomes isolation and identification**

When the cell confluence reached 70%, the cell culture medium was replaced with complete medium that consists of basal culture medium added with 10% exosome-depleted FBS. After 48 h, the cell culture supernatant was collected and centrifuged at 300 g for 10 minutes, followed by 2,000 g for 20 min and 10,000 g for 30 min to remove cellular debris and large vesicles. After filtering the supernatant through a 0.22 μm membrane, the supernatant was centrifuged at 140,000 g for 90 min at 4°C using ultracentrifuge (Beckman Coulter, CA, USA). The pellet was resuspended in 30 mL of PBS, followed by another round of ultracentrifugation at 140,000 g for 90 min at 4°C. After discarding the supernatant, the pellet was resuspended in 200 μL of PBS and stored at -80°C. The isolation of plasma exosomes was performed according to the manufacturer's instruction (No. UR52151, Umibio, Shanghai, China). The size of exosomes was measured using ZetaView (Particle Metrix, München, Germany). Additionally, the morphology of exosomes was recorded using transmission electron microscopy (TEM, Hitachi, Japan).

**Exosomes labelling and tracking**

Exosomes were labeled with PKH67 (Umibio, Shanghai, China) according to manufacturer’s instructions. After co-culturing the labeled exosomes with macrophages at 37°C for 12 h, the F-actin of the macrophages was stained with YF^®^555-phalloidin (UElandy, Suzhou, China). Then, images were captured using a fluorescence microscope (Zeiss, Germany). To ascertain the transfer of exosomal miR-106a-5p, exosomes were extracted from CRC cells transfected with Cy3-labeled miR-106a-5p. These exosomes were incubated with macrophages for 24 h, following by immunofluorescence assay.

**Western blot**

After lysing the cells or exosomes with RIPA lysis buffer, loading buffer was added to the lysate and the mixture was boiled to denature the proteins. Then, proteins were resolved by SDS-PAGE gel electrophoresis, and transferred onto the PVDF membrane (0.45μm, Millipore, USA). The PVDF membranes were blocked with 5% skim milk for 2 h and then incubated overnight with the primary antibody at 4°C. Then, the membranes were incubated 2 h with horseradish peroxidase-conjugated secondary antibody at room temperature. Finally, chemiluminescence was executed using ECL reagent (Tanon, Shanghai, China). The antibodies are exhibited in Table S2.

**RNA extraction, reverse transcription PCR and qRT-PCR**

Total RNA was extracted by TRIzol Reagent (Takara, Kusatsu, Japan). Then, miRNA was reverse transcribed to cDNA using microRNA Reverse Transcription Kit (EZBioscience, Roseville, USA) and mRNA was similarly reverse transcribed to cDNA employing the Hifair^®^ Ⅲ 1st Strand cDNA Synthesis SuperMix for qPCR (Yeasen, Shanghai, China). Then, qRT-PCR was conducted using EZ-Probe qPCR Master Mix for microRNA kit (EZBioscience, Roseville, USA) and Hieff^®^ qPCR SYBR Green Master Mix kit (Yeasen, Shanghai, China). U6 and GAPDH were used as internal reference genes to calculate a 2-ΔΔCt value. cel-miR-39 was used as exogenous reference genes. All primer information is exhibited in Table S1.

**Enzyme linked immunosorbent assay (ELISA)**

The concentration of IL-10 and TGF-β produced by macrophages were detected by IL-10 and TGF-β ELISA kits (FineTest, Wuhan, China), following the manufacturer’s instructions.

**Flow cytometry**

Macrophages were prepared into a single-cell suspension and Fc receptors were blocked using human BD FC block (BD Pharmingen) prior to staining. Then, the macrophages were washed twice with flow buffer, and fixed with fixation solution (BioLegend, California, USA) at room temperature for 30 min, followed by centrifugation and then washed twice again with flow buffer. The macrophages were then resuspended in 100 µL of intracellular staining permeabilization wash buffer (BioLegend, California, USA), and incubated with human anti-CD206 at 4°C for 1 h, followed by centrifugation and then washed twice again with flow buffer. Subsequently, the cells were incubated with fluorescently labeled secondary antibody in the dark at 4°C for 30 min. Finally, after centrifugation and washed twice with flow buffer, the macrophages were resuspended in 100 µL of flow buffer for analysis. Flow cytometry was executed with a NovoCyte D3000 flow cytometer (Agilent, California, USA). The antibodies are exhibited in Table S2.

**RNA-binding protein immunoprecipitation (RIP) assay**

RIP assay was conducted using a Magna RIP^TM^ Kit (Millipore, USA), according to the manufacturer’s instruction. Briefly, cells or exosomes were lysed using complete RIP lysis buffer (RIP lysis buffer added with protease inhibitor cocktail and RNase inhibitor). Then, the RIP lysate was incubated with beads-antibody complex (anti-hnRNPA1 or IgG) overnight at 4 ℃. After centrifuging and washing the magnetic beads six times, RNA purification and extraction were performed. The isolated RNA was reverse transcribed and performed qRT-PCR. The calculation method for the relative quantification data of RIP-PCR was based on the manufacturer's instructions. The antibodies are exhibited in Table S2.

**Biotin miRNA pulldown assay**

Cellular nuclear and cytoplasmic proteins were extracted using NE-PER™ nuclear and cytoplasmic extraction reagents (Thermo Scientific, USA). Exosomes protein was extracted by lysis buffer complex (lysis buffer added with protease inhibitor and RNase inhibitor). The biotin labeled miR-106a-5p or mutated miR-106a-5p oligonucleotides was incubated with the above lysates overnight at 4 ℃. In the above lysates, blocked streptavidin magnetic beads (BersinBio, Guangzhou, China) were added and incubated with rotation at 4 °C for 4 h. After washing the beads five times with washing buffer, the proteins bound to the beads were eluted by protein elution buffer. Finally, loading buffer was added and boiled for western blot analysis.

**Dual-Luciferase reporter gene assay**

All pmiR-GLO plasmids were purchased from Miaoling Bioscience & Technology (Wuhan, China). Based on the manufacturer's protocol, the dual-luciferase reporter assay was conducted (Yeasen, Shanghai, China). Fluorescence signals were detected by Spark (TECAN, Mannedorf, Switzerland).

**Transwell assay**

CRC cells migration and invasion ability were assessed by transwell chambers (8 μm; Corning, USA) with or without Matrigel (Corning, USA). The detail protocol of transwell assay was performed as our previously described[2].

**Animal experiments**

This study’s animal experimental protocols approval from the Animal Ethics Committee of the First Affiliated Hospital of Nanchang University. All male BALB/c nude mice were acquired from Charles River Biotechnology (Zhejiang, China). In establishing the liver metastasis model via splenic, each mouse was intrasplenically injected with 2 million luciferase-lentivirus-transfected LoVo cells that were previously incubated in different CM. After four weeks of injection, in vivo imaging was performed on the mice using IVIS® Lumina III (PerkinElmer, MA, USA). Subsequently, the mice were sacrificed, and liver specimens were harvested for photography and HE staining.

**References:**

[1] Y. Hao, S. Hao, E. Andersen-Nissen, W.R. Mauck, S. Zheng, A. Butler, et al., Integrated analysis of multimodal single-cell data. Cell 184 (2021) 3573-3587.e29.

[2] T. Li, C. Tang, Z. Huang, L. Yang, H. Dai, B. Tang, et al., miR-144-3p inhibited the growth, metastasis and epithelial-mesenchymal transition of colorectal adenocarcinoma by targeting ZEB1/2. Aging (Albany NY) 13 (2021) 17349-17369.
