# Supplementary material for: Exosomal miR-106a-5p from highly metastatic colorectal cancer cells drives liver metastasis by inducing macrophage M2 polarization in the tumor microenvironment
Source: J Exp Clin Cancer Res. 2024 Oct 9;43:281. doi: 10.1186/s13046-024-03204-7 (PMC11462797; doi:10.1186/s13046-024-03204-7)

**Supplementary Fig1(Fig S1)**


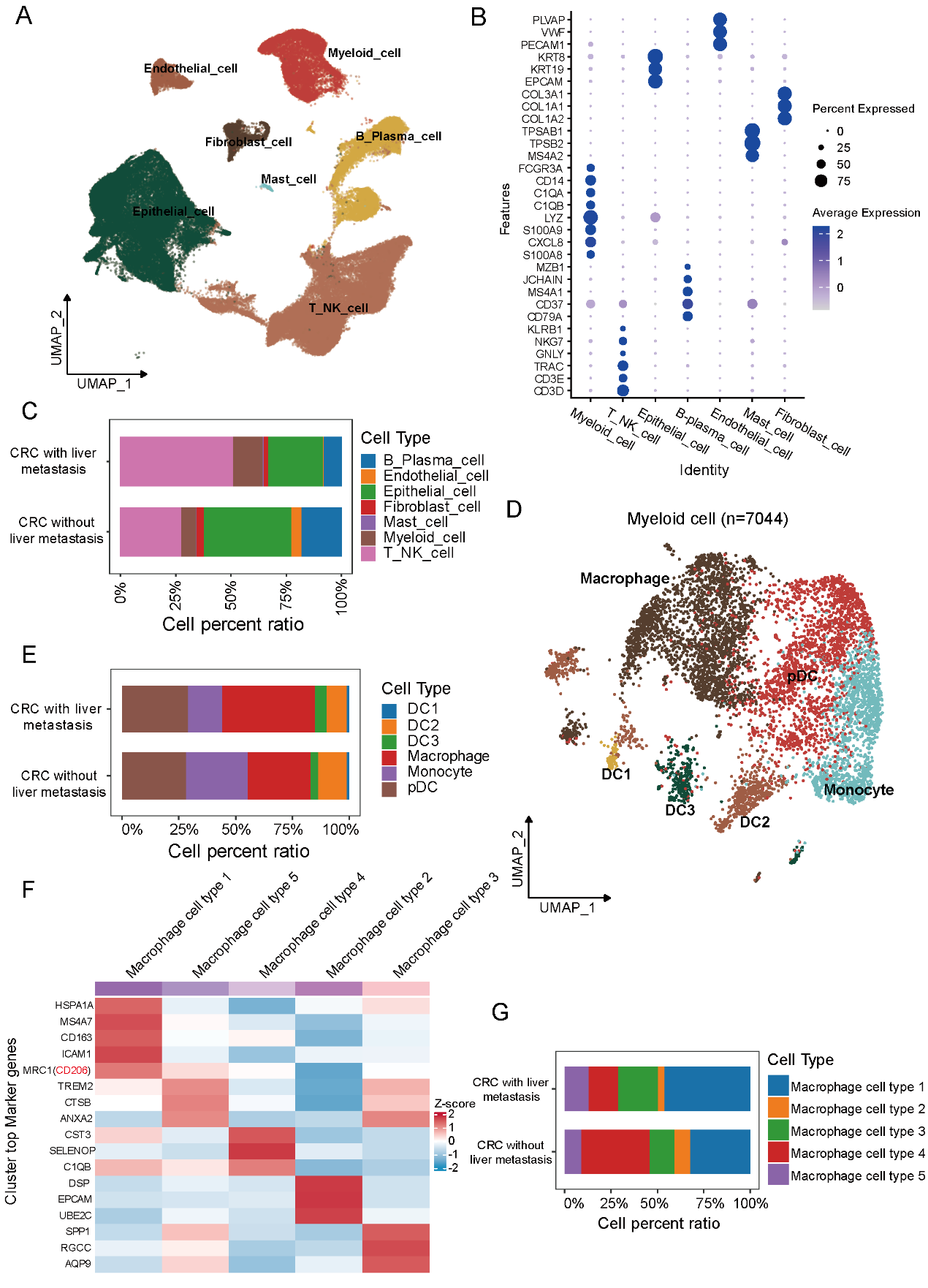


**Supplementary Fig2(Fig S2)**


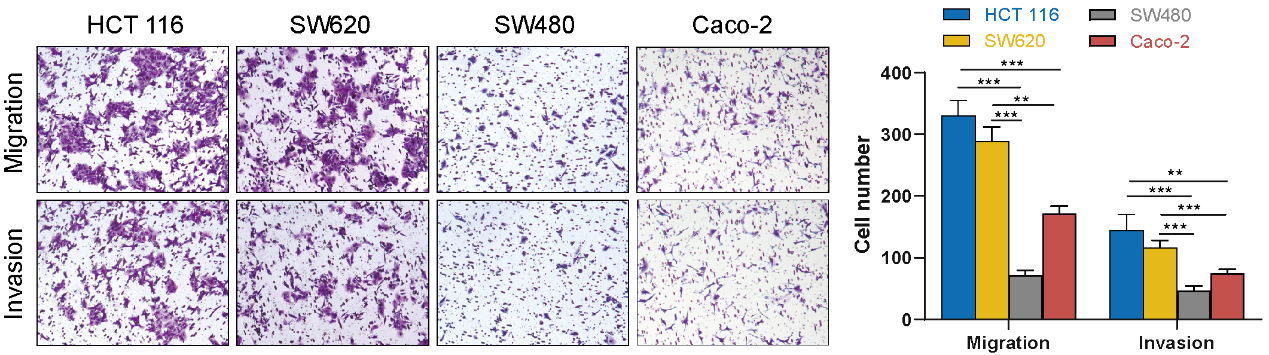


**Supplementary Fig3(Fig S3)**
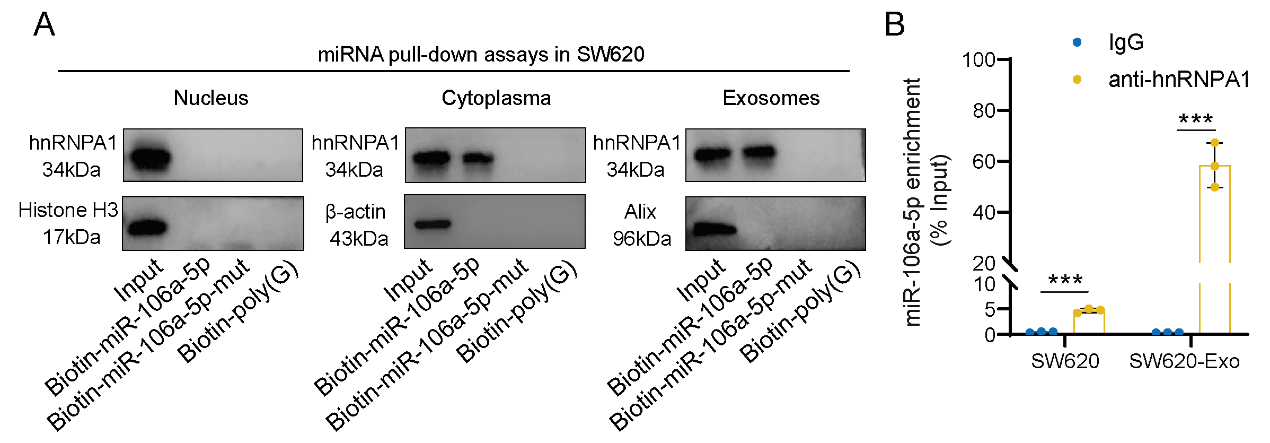


**Supplementary Fig4(Fig S4)**


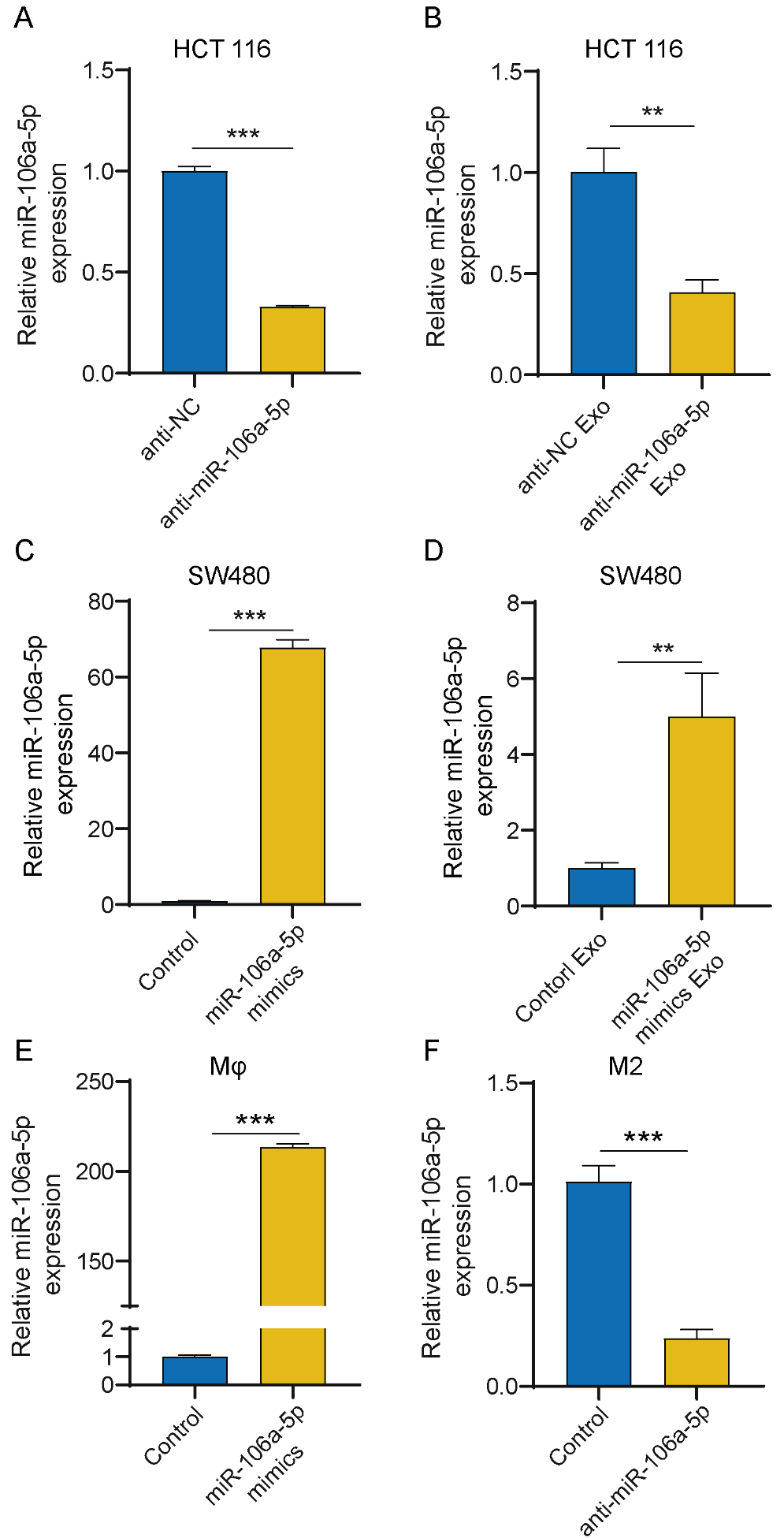


**Supplementary Fig5(Fig S5)**


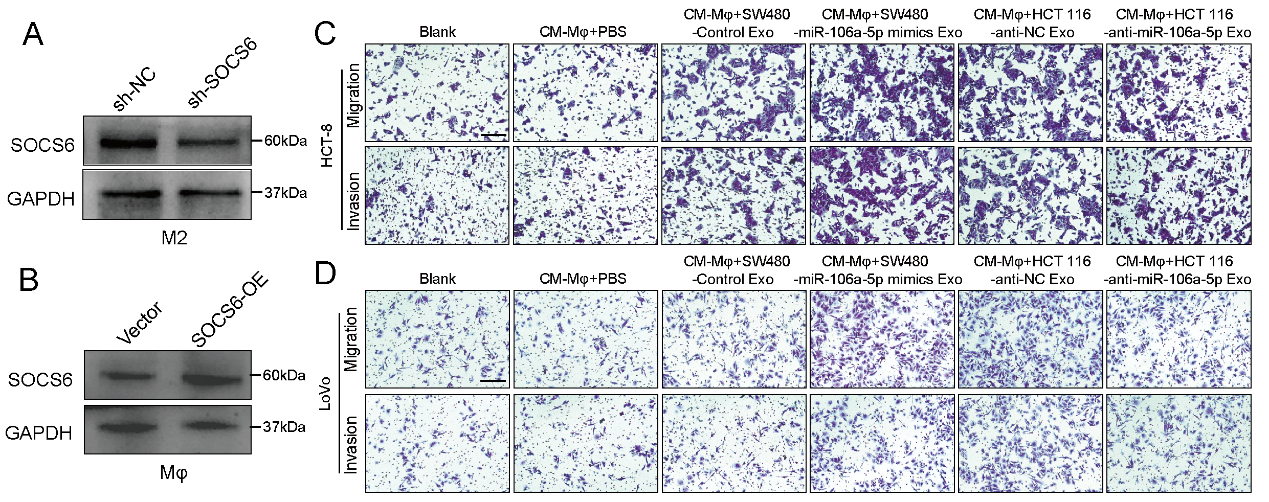

Supplement: Supplementary file 6 — Supplementary Material 6: Fig. S1 Single-cell expression atlas of CRC with or without liver metastasis. (A) Uniform manifold approximation and projection (UMAP) plot of the major cell types in all samples (n = 13). (B) Dot plot exhibiting the marker genes across the cell types. (C) Histogram showed the proportions of cell types in CRC tissues with or without liver metastasis. (D) UMAP plot of myeloid clusters. (E) Histogram showed the proportions of myeloid types in CRC tissues with or without liver metastasis. (F) Heatmap exhibiting the marker genes across macrophage subsets. (G) Histogram showed the proportions of macrophage types in CRC tissues with or without liver metastasis, Fig. S2 Transwell assay was conducted to explore the metastatic capability of four different CRC cells. ** p < 0.01, *** p < 0.001, Fig. S3 (A) Western blot was employed to assess hnRNPA1 expression in samples obtained from miRNA pulldowns, utilizing nuclear, cytoplasmic, or exosomal lysates from SW620 cells. (B) RIP assays were conducted using an anti-hnRNPA1 antibody (or IgG as a control) on lysates derived from SW620 cells or exosomes. qRT-PCR was employed to quantify miR-106a-5p levels in the immunoprecipitated samples, expressed as percentages relative to the input (% input). *** p < 0.001, Fig. S4 (A) qRT-PCR was performed to confirm the efficiency of lentivirus-mediated knockdown of miR-106a-5p in HCT 116 cells. (B) qRT-PCR was performed to detect the expression of miR-106a-5p in the exosomes of HCT 116 cells treated with miR-106a-5p knockdown lentivirus and lentiviral control. (C) qRT-PCR was performed to confirm the efficiency of lentivirus-mediated overexpression of miR-106a-5p in SW480 cells. (D) qRT-PCR was performed to detect the expression of miR-106a-5p in the exosomes of SW480 cells treated with miR-106a-5p overexpression lentivirus and lentiviral control. E-F qRT-PCR was performed to detect the expression of miR-106a-5p in Mφ cells after transfection with miR-106a-5p mimic [file 13046_2024_3204_MOESM6_ESM.docx]
